# Alternative Proteins for Fish Diets: Implications beyond Growth

**DOI:** 10.3390/ani12091211

**Published:** 2022-05-07

**Authors:** Cláudia Aragão, Ana Teresa Gonçalves, Benjamín Costas, Rita Azeredo, Maria João Xavier, Sofia Engrola

**Affiliations:** 1 Centre of Marine Sciences (CCMAR), Universidade do Algarve, 8005-139 Faro, Portugal; mmxavier@ualg.pt (M.J.X.); sengrola@ualg.pt (S.E.); 2 GreenCoLab—Associação Oceano Verde, Universidade do Algarve, 8005-139 Faro, Portugal; anagoncalves@sparos.pt; 3 SPAROS Lda, 8700-221 Olhão, Portugal; 4 Interdisciplinary Centre of Marine and Environmental Research (CIIMAR), 4450-208 Matosinhos, Portugal; bcostas@ciimar.up.pt (B.C.); mleme@ciimar.up.pt (R.A.); 5 School of Medicine and Biomedical Sciences (ICBAS-UP), Universidade do Porto, 4050-313 Porto, Portugal

**Keywords:** aquaculture, fish diet formulations, fishmeal replacement, alternative protein sources, intestinal health, microbiota, immune response, disease resistance, stress response, oxidative stress

## Abstract

**Simple Summary:**

Aquaculture is now well-established as a provider of protein for human consumption, and its contribution will be paramount to providing food for a nine billion population in 2050. Protein is usually the major constituent of fish feeds and the most expensive ingredient. For years, fishmeal was the preferential protein source in fish diets, but environmental and economic concerns led to the search for more sustainable proteins. Hence, research on alternative protein sources to fishmeal was fruitful, being firstly directed to terrestrial plant ingredients. Recently, research on novel ingredients, such as insect meals, macroalgae, microalgae, and yeasts, has proliferated. However, the impacts of protein and its constituents (amino acids) go beyond fish growth. Thus, this review will provide knowledge on the impacts of alternative/novel protein sources on fish stress and immune responses, disease resistance, and health. Although some negative impacts of alternative ingredients, for instance, on gut integrity and immune responses have been observed, research results also point to the potential beneficial effects of novel ingredients, such as insect meals, on fish health. This information is essential to the development of innovative diets that guarantee the production of healthy fish with high quality standards and optimised welfare conditions.

**Abstract:**

Aquaculture has been challenged to find alternative ingredients to develop innovative feed formulations that foster a sustainable future growth. Given the most recent trends in fish feed formulation on the use of alternative protein sources to decrease the dependency of fishmeal, it is fundamental to evaluate the implications of this new paradigm for fish health and welfare. This work intends to comprehensively review the impacts of alternative and novel dietary protein sources on fish gut microbiota and health, stress and immune responses, disease resistance, and antioxidant capacity. The research results indicate that alternative protein sources, such as terrestrial plant proteins, rendered animal by-products, insect meals, micro- and macroalgae, and single cell proteins (e.g., yeasts), may negatively impact gut microbiota and health, thus affecting immune and stress responses. Nevertheless, some of the novel protein sources, such as insects and algae meals, have functional properties and may exert an immunostimulatory activity. Further research on the effects of novel protein sources, beyond growth, is clearly needed. The information gathered here is of utmost importance, in order to develop innovative diets that guarantee the production of healthy fish with high quality standards and optimised welfare conditions, thus contributing to a sustainable growth of the aquaculture industry.

## 1. Introduction

Aquaculture is now well-established as an animal protein provider for human consumption. Aquaculture production has surpassed beef production in 2013 [1] and, in 2018, provided more than 50% of the fish for human consumption [2]. The growth and establishment of aquaculture in the last decades cannot be disassociated with the advances in fish nutrition [3].

Protein is the most expensive nutrient in fish diets, and inclusion levels usually range from 30 to 50%. For decades, fishmeal was used as the main protein source in fish diets, due to its high protein content and balanced amino acid profile [4]. Furthermore, its high palatability, high nutrient digestibility, and general absence of antinutritional factors have contributed to the definition of fishmeal as a golden standard [5,6]. However, sustainability concerns at the environmental, social, and economical levels lead to increasing research towards fishmeal replacement strategies [3]. The urge to find alternative protein sources to fishmeal gained a significant importance in the beginning of this century, and a substantial research effort has been dedicated to finding alternative proteins, focusing essentially on terrestrial plant ingredients. Still, most plant protein sources contain a variety of biologically active antinutritional factors, which may negatively affect the feed intake, digestion and absorption of nutrients, and fish health status [4,5,6].

The continuous growth in aquaculture production has posed some extra pressure on the use of terrestrial plant ingredients. In this sense, research on alternative proteins sources for fish diets has continued beyond terrestrial plant ingredients. Rendered animal by-products, such as poultry by-product meal, blood meal, and feather meal are valuable ingredients that contribute to circular economy, thus improving the sustainability of the sector. Although their use in Europe was limited from 2001 to 2013, due to concerns with the transmissible spongiform encephalitis, these protein sources have been utilised in aquaculture diet formulations with considerable success [4,7].

Research on novel ingredients for fish feeds, such as microalgae and other single cell proteins, macroalgae, and insect meals, has proliferated recently and will continue to expand [3]. Macro- and microalgae have been considered alternative ingredients, due to their high growth rates and non-competition of arable lands for cultivation. Macroalgae encompass algae that are multicellular and macroscopic and, depending on the species, their nutritional value is quite different. Although several species have been considered alternative protein sources for fishmeal, the growing interest on their use as a source of bioactive compounds and prebiotics has led to increased research (see reviews on the subject [8,9,10]). Microalgae integrate the group of single cell proteins, together with yeasts and bacterial meals, although for the purposes of the present review microalgae are considered together with macroalgae. Due to their nutritional value and beneficial properties, microalgae have been recognised as an interesting ingredient for aquafeeds, and their potential as an alternative protein source has been acknowledged [11,12,13,14,15]. Their high production costs have been a major constraint for viable inclusion in aquafeeds; however, in the near future, this limitation is expected to be surpassed [14]. Single cell proteins are isolated from the cells of microorganisms with high protein content, such as fungi and bacteria. In recent years, these dried or processed biomasses have shown their potential as a replacement for fishmeal and terrestrial plant proteins, especially on what concerns yeasts (see recent reviews on the subject [16,17,18]).

The utilization of insect meal for fishmeal and plant protein replacement in aquaculture has been recently addressed by many researchers. Insects represent a sustainable ingredient with a very interesting nutritional profile and, since their production can be based on substrates that comply with the principles of circular economy (e.g., food wastes), they have become one of the main focuses in fish nutrition over the last five years (see reviews [19,20,21,22,23,24,25] for further information on this subject). Indeed, insects are considered an appropriate protein source, with the black soldier fly (*Hermetia illucens*) and yellow mealworm (*Tenebrio molitor*) as the most important candidate species. Hence, scientific knowledge on novel protein sources is expected to significantly increase in the near future, contributing to their successful implementation in fish diet formulations.

Research in the last decades has demonstrated that the importance of protein and its constituents (amino acids) goes beyond growth. Although alternative protein sources are needed, contemporary research has identified that fishmeal replacement may result in adverse effects in fish physiology, metabolism, and health [3,4]. Considering the expected increase in aquaculture production to feed the growing population, special attention should be given to these aspects, thus ensuring that this industry keeps growing as a sustainable activity. Dietary proteins are the main source of amino acids; apart from their known role in protein synthesis, growth, energy production, and as substrates for key metabolic pathways, they have been recognised as functional ingredients for fish health and welfare [26,27,28]. Additionally, protein hydrolysates gained more attention as a functional ingredient to promote animals’ health and provide additional value to this new generation of aquafeeds [29]. Functional ingredients and additives assume an increased importance in the actual context of fish feed formulation, where levels of fishmeal inclusion have been decreased and novel proteins are being tested on a regular basis.

In this context, this work intends to comprehensively review the impacts of these alternative and novel dietary protein sources on fish stress and immune responses, health, and disease resistance. This information is of utmost importance to the development of innovative diets that guarantee the production of healthy fish with high quality standards and optimised welfare conditions.

## 2. Impacts of Alternative Protein Sources on Fish Intestinal and Hepatic Health

In the past two decades, an intense research effort has been devoted to unlocking the potential of plant-derived ingredients, as an alternative to gradually replace traditional ingredients of marine origin (fishmeal and fish oil), in aquafeeds formulation [3,5]. Earlier, soybean was considered a good alternative to fishmeal, due to its high protein content, relatively balanced amino acid profile, and availability [6]. However, the first reports showed that dietary inclusion of soybean meal at levels above 10% induced enteritis in salmonids [30]. The disturbance was associated with the presence of antinutritional factors in crude soybean meals, especially soya saponins [31]. High levels (±20%) of dietary soybean meal inclusion are still reported to induce enteritis in Atlantic salmon (*Salmo salar*) and rainbow trout (*Oncorhynchus mykiss*) [32,33,34]; further research has shown that salmonids were not the only fish affected. Signs of soybean-induced enteritis were also recently reported in orange-spotted and hybrid groupers (*Epinephelus coioides* and *E. fuscoguttatus* × *E. lanceolatus*) [35,36], yellowtail (*Seriola dorsalis*) [37], Japanese seabass (*Lateolabrax japonicus*) [38,39], and totoaba (*Totoaba macdonaldi*) [40].

The integrity of the intestinal epithelium is fundamental to secure nutrient absorption, and the negative effects of plant protein-based diets in the intestinal health may lead to disturbances in the intestinal absorptive function. In meagre (*Argyrosomus regius*) juveniles that were fed a diet containing more than 50% of plant ingredients for 10 weeks, the intestinal epithelium showed a secretory function, instead of its normal absorptive function, indicating a pathological state of diarrhoea [41]. This intestinal disturbance has been previously reported in Atlantic salmon presenting soybean-induced enteritis [42,43]. The alterations in the intestinal epithelium, due to enteritis, negatively affect nutrient digestibility, intestinal enzyme activities, and the expression of amino acid and peptide transporter genes [38,40]. Therefore, the disturbances found at intestinal epithelial level are translated in a lower nutrient availability for fish, which, in some circumstances, may be reflected in a reduction in growth performance.

Besides the important role of the intestine in nutrient absorption, intestine integrity is essential in the defence against pathogens. Japanese seabass, when fed soybean-based diets (>30% of inclusion) for eight weeks, showed increased intestinal permeability and impaired intestinal mucosal barrier function [38]. Soya saponins, per se, have been shown to increase intestinal epithelial permeability in Atlantic salmon after feeding fishmeal-based diets with soya saponin inclusion for 53 days, even if not inducing enteritis [42]. It should be noted that not only soybean-based diets induce disturbances at intestinal level. Diets with high levels of novel ingredients, such as insect meals, microalgae, macroalgae, and yeast (*Saccharomyces cerevisiae*), have also shown to affect the fish intestinal barrier function. In Artic charr (*Salvelinus alpinus*) and gilthead seabream (*Sparus aurata*) that were fed diets with these alternative ingredients for 80 or 100 days, respectively, an increased paracellular permeability was observed [44,45]. The compromised intestine integrity enhances the risk of the intrusion of pathogens or exposure to foreign antigens from the gut lumen, thus increasing disease susceptibility and/or leading to an inflammatory reaction. Additionally, given the important role of the intestine in the immune function, the inflammatory reactions observed at the intestinal level in fish fed alternative diets may result in an impaired immunological response.

Soybean-induced enteritis has been the subject of many studies on fishmeal replacement strategies, but the other antinutritional factors that are present in plant ingredients may also affect fish health. For instance, carob seed germ meal contains antinutritional factors, primarily tannins. Based on the morphological alterations found in the liver and distal intestine of gilthead seabream juveniles, a possible negative effect of long-term feeding with diets containing more than 34% of carob seed germ meal was suggested [46]. Lupin meal is another plant protein source that also contains antinutritional factors (phytic acid, tannins, saponins, and lectins). Barramundi (*Lates calcarifer*), when fed diets with 75% replacement of fishmeal by lupin meal (51% of dietary inclusion) for 60 days, developed liver steatosis and necrosis in the kidney, resulting in lower growth performance than in fish that were fed fishmeal-based diets [47]. Even when using plant proteins that have relatively low levels of antinutritional factors, the impacts on fish health may be found. For example, a high dietary level of wheat gluten (30% of inclusion) can have a negative impact on the intestinal and liver health of Atlantic salmon after feeding for nine weeks, with symptoms similar to gluten sensitivity in humans [48].

The technical processing steps in ingredient and diet production, such as alcohol extraction, fermentation, or heat, may inactivate or reduce the content of the antinutritional factors in plant ingredients [4]. For instance, soybean-induced enteropathy was not observed in Atlantic salmon that were fed diets with up to 25% of soy protein concentrate for 56 days [32]. Additionally, inclusion of up to 30% of bioprocessed soybean meal affected neither growth performance nor intestinal health in rainbow trout after feeding for 125 days [49]. However, highly processed plant proteins are expensive and high inclusion levels are not economically feasible in production scenarios.

Another successful strategy to counteract intestinal inflammation is the use of dietary supplements. Functional additives, comprising of a mixture of antioxidant, immunopotentiator, and trace elements, were able to partially eliminate soybean-induced enteritis in Japanese seabass [39]. The addition of probiotics (*Lactobacillus fermentum* and *Lactobacillus plantarum*) to soybean meal-based (20% of inclusion) diets did not completely prevent the development of enteritis in Atlantic salmon, but enhanced intestinal health [34]. Supplementation with butyrate (0.2% of inclusion) improved gut morphology and mitigated the symptoms of inflammation in European seabass (*Dicentrarchus labrax*), black seabream (*Acanthopagrus schlegelii*), and largemouth bass (*Micropterus salmoides*) that were fed high soybean meal (±30% inclusion) diets [50,51]. Butyrate seems to be used as a major energy source by epithelial cells of the intestine, thus stimulating cell proliferation and differentiation upon injury [51]. Dietary taurine supplementation (0.2%) also normalised the intestinal abnormalities and reduced the intestinal inflammation observed in European seabass that were fed soy protein (16.7% soybean meal and 12.8% full-fat soy) diets [50].

The search for novel ingredients to fishmeal is still in its infancy. Alternative protein sources, other than plant ingredients, may exert obvious impacts on fish health, inducing modifications at the intestinal and hepatic levels. The inclusion of more than 7.5% of whey protein concentrate in Nile tilapia (*Oreochromis niloticus*) diets resulted in swollen hepatocytes and congested hepatic blood vessels after 10 weeks of feeding [52]. This translated to disturbances in the liver function, leading to increased plasma levels of the metabolic enzymes—alanine aminotransferase (ALT) and aspartate aminotransferase (AST)—which is an indicator of liver disease. In European seabass, the inclusion of more than 16% of whey protein in diets increased the activity of liver function enzymes and disrupted kidney function after 10 weeks of feeding; although, only at 27% of dietary inclusion, histopathological signs in intestine and liver tissues were observed [53]. Insect meals are also being increasingly used as novel protein sources for fish diets. In rainbow trout, fishmeal replacement by black soldier fly prepupae meal resulted in a reduction in the absorptive epithelial surface, as well as the potential occurrence of gut inflammation [54]. In studies with juvenile barramundi, histological alterations at the intestinal epithelial level were reported after feeding totally replaced fishmeal diets (69.5% of poultry by-product meal inclusion or 63% of poultry by-product meal and 12% of insect meal) for six weeks, thus lowering the digestion and absorption surface area and impairing growth performance [55,56]. Furthermore, in these fish, increased serum levels of AST and glutamate dehydrogenase were found, associated with hepatic multifocal necrosis. Other studies also reported hepatic disturbances in fish that were fed diets containing rendered animal proteins. The inclusion of more than 24% of an animal protein blend (poultry by-product, meat and bone, spray-dried blood, and hydrolysed feather meals) induced hepatic steatosis in Japanese seabass after eight weeks of feeding [57]. In hybrid grouper, the inclusion of a blend of rendered animal proteins (poultry by-product, shrimp, and spray-dried blood meals), at levels replacing 80% of dietary fishmeal (57% inclusion level), induced hepatic steatosis by modulating the lipid metabolism-related genes and inducing hepatocyte apoptosis via the up-regulation of apoptosis-related genes [58]. Additionally, in hybrid grouper, high levels of dietary poultry meal inclusion (>31% of the diet) induced steatosis in fish hepatocytes after eight weeks of feeding [36]. The authors related this result with the high lipid level and almost no EPA (eicosapentaenoic acid) or DHA (docosahexaenoic acid) found in poultry meal. These results serve as a reminder that the ingredients used as alternative protein sources to fishmeal are more than just protein. Hence, the processing of alternative ingredients to improve its nutritional value may be paramount, and further research is clearly necessary.

The search for sustainable alternative ingredients that promote fish health and welfare is clearly well-established as one of the priorities for aquaculture. An interesting strategy on the use of novel ingredients is its utilization for counteracting the negative effects of some plant proteins. For instance, no hepatic damage was observed in barramundi that were fed high lupin meal diets (51% of inclusion), when 4.6% of tuna fish protein hydrolysates were included, contrary to what was observed in fish that were fed non-supplemented diets [47]. Additionally, dietary inclusion of protein hydrolysates (from krill, shrimp, or tilapia; circa 3% of inclusion) in low fishmeal (plant protein-based) diets improved gut macromorphological aspects and enhanced the apparent digestibility of dry matter and crude protein in olive flounder (*Paralichthys olivaceus*) after 11 weeks of feeding [59]. The inclusion of single cell proteins in plant protein-based diets has also provided interesting results. The inclusion of bacterial meal, of the microalgae *Chlorella vulgaris* or of the yeast *Candida utilis*, in diets with 20% of soybean meal, was highly effective in counteracting soybean-induced enteropathy in Atlantic salmon [60,61]. Oppositely, the yeast *S. cerevisiae* had no functional effects in Atlantic salmon diets [60]. However, in rainbow trout, the inclusion of a protein-rich yeast fraction (from *S. cerevisiae*) in plant protein diets, totally devoid of fishmeal, resulted in a larger fish internal intestinal surface area, which contributed to an enhanced nutrient absorption capacity [62]. Furthermore, while rainbow trout that were fed the 100% plant protein diet showed signs of moderate enteritis in the distal intestine, this inflammation was gradually alleviated with increasing incorporation of the yeast fraction in the diet (up to 15% of inclusion). As for the microalgae, the dietary inclusion of a blend of dried marine *Tisochrysis lutea* and *Tetraselmis suecica* biomass improved the gut digestive and absorptive functions of European seabass, relative to fish given a soybean meal-rich diet (35% of inclusion) for 105 days [63]. Other novel protein sources, such as insect meals, have also shown potential to ameliorate intestinal inflammation. Total replacement of fishmeal in plant protein-based diets by black soldier fly larvae meal was associated with lower enterocyte steatosis in the proximal intestine of Atlantic salmon [64]. The inclusion of defatted black soldier fly or poultry by-product meals, in diets totally devoid of fishmeal, as a replacement or a complement of plant protein-rich ingredients, resulted in improved growth and gut health in gilthead seabream and rainbow trout [65,66]. Interestingly, the combination of novel protein sources has also been shown to translate to positive impacts for fish metabolism and health. An increase in the inclusion level of full-fat black soldier fly larvae meal (35% of inclusion) in poultry by-product meal diets (totally devoid of fishmeal) resulted in no obvious hepatic lesions in barramundi [67], contrary to what has been previously observed with poultry by-product meal diets, without [55] or with 12% of full-fat black soldier fly larvae meal inclusion [56]. Thus, the dietary inclusion of alternative ingredients, such as poultry and insect meals, seems a promising strategy for counteracting the negative side effects that might be observed in non-fishmeal diets.

## 3. Impacts of Alternative Protein Sources on Fish Microbiota

Fish gut microbiota is considered a regulator of fish health [68,69], participating in key processes such as digestion, defence and immune responses, and tissue stability, among others [70,71]. One of the most relevant processes intervened by microbiota is the digestion of feed components that are indigestible for the host, leading to the synthesis of short chain fatty acids [72]. These represent a major energy source for intestinal epithelial cells and are essential for gut health. Microbial composition has been widely studied for different species and is known to vary with species, season, and life cycle events, among other factors (see the recent reviews on the subject [68,73,74]). Fish gut microbiota is also quite sensitive to dietary manipulations [75,76], and dietary modulation of its composition will change the microbiome function, so the fish will accommodate a response with physiological consequences. Most studies focusing on fish gut microbiome modulation through diet assessed the impact of functional ingredients, such as pre- or probiotics [75,77]. Indeed, alternatives to fish- and plant meals as protein sources, such as algae biomass or hydrolysates, often have functional properties when included at lower dosages [78,79] and are associated with shifts in microbial community structure. However, this section will focus on the effects of the alternative protein sources that are applied as a replacement for more classical ingredients (i.e., fishmeal and soybean meal (or concentrates)), thus reviewing studies where these ingredients were included at a minimum of 5% (dry matter basis).

Protein is the most important bulk nutrient, and it modulates fish gut microbiota [68,80,81]. Depending on the source, within the alternative proteins, the hydrolysates, algae, and yeasts are expected to produce alterations in the microbiota community composition. The first have short peptides that are used as substrate by several intestinal microorganisms and modulate the interactions between the microbiota and host’s enterocytes [82,83]. The latter have been used as probiotics, and they are rich in compounds that can act as prebiotics, such as mannan oligosaccharides [84]. Micro- and macroalgae, however, have been highlighted as prebiotic ingredients [85,86], as well as a source of micronutrients and minerals, essential for the gut well-functioning and stability. As alternative protein sources, studies have shown controversial results, where efficiency depends greatly on inclusion rate, algae species, and biomass pre-treatments that will potentiate digestibility and nutrient utilization. Regarding algae’s ability to modulate gut communities’ diversity and richness (Table 1), results were varied and seemed to depend on fish and algae species. Feeding European seabass a diet with 15% inclusion of the cyanobacteria *Arthrospira* (formerly *Spirulina*) *platensis* biomass for 93 days reduced the feed conversion ratio (FCR) and resulted in an unfavourable growth performance [87]. No differences in gut community richness and diversity were observed; however, it was evident that there was a reduction of the abundance of several members of Proteobacteria phylum, as well as an increase of the abundance of the genera *Persicirhabdus*, *Methylobacterium*, *Acinetobacter*, and *Sediminibacterium*. In a study with Atlantic cod (*Gadus morhua*) juveniles, no significant differences were found in gut microbiota or growth performance when fish were fed a diet with 10% *Ulva rigida* biomass in a 12-week trial [88]. On the other hand, in the same study, a 10% inclusion of the microalgae *Ascophyllum nodosum* reduced growth concomitantly with a reduced gut microbiota diversity and higher abundance of the genera *Psychromonas*, *Propionigenium*, and *Clostridium*. Since the first has been linked to nutritional compensation for unbalanced diets and the second is known to degrade cellulose and complex carbohydrates, as well as to produce anti-inflammatory products from its metabolism [89], the authors suggested a possible microbial modulation to compensate for any deleterious effects of the algae inclusion in the diet. When gilthead seabream juveniles were fed 10% *Tetraselmis chuii* or *Phaeodactylum tricornutum* biomass for 30 days, intestinal microbial diversity and richness were reduced, although no differences were observed in growth performance [90]. Despite those studies, others have reported positive alterations of the gut microbiota diversity when algae are used as a protein source. Feeding rainbow trout juveniles diets with 5% *Schizochytrium limacinum* biomass increased gut microbial diversity and induced a higher abundance of lactic acid bacteria (LAB) after 15 weeks [91]. An increase in microbial diversity was also found in juvenile hybrid grouper fed diets with 5 or 30% *Arthrospira platensis* biomass, associated with higher growth at the lowest inclusion level [92]. However, the most promising results have been shown when using macroalgae as alternative protein source. Feeding Senegalese sole (*Solea senegalensis*) a diet with 5% inclusion of *Ulva ohnoi* for 45 days resulted in higher microbial diversity in the anterior intestine [93]. Members of the genus *Vibrio* increased their abundance in the macroalgae-fed group; however, *Tenacibaculum*, one of the most relevant pathogens in aquaculture, had a lower abundance. A 15% dietary inclusion of *Ulva rigida* biomass increased richness in the gilthead seabream gut, but a higher inclusion (25%) had an opposite effect after 70 days [94]. Interestingly, when gilthead seabream juveniles were fed the same 25% of *Ulva rigida*, but for less time (30 days), gut bacterial communities were richer, and an increase in abundance of the genus *Lactobacillus* was observed, as well as a reduction of *Photobacterium* [95]. Indeed, the ability of macroalgae to modulate gut microorganisms has been acknowledged (see review [96]), and it assumes a special relevance in aquaculture, since fish share the environment with opportunistic microorganisms that easily become pathogenic. As in the above-mentioned study, the dietary inclusion of *Gracilaria cornea* biomass, as a protein source for 70 days, reduced the abundance of *Vibrio* and diminished *Photobacterium* genus members to a residual abundance in the gilthead seabream’s intestine [94]. A reduction in the abundance of pathogenic organisms might result in the observed increased disease resistance that fish have when fed macroalgae. This was observed by some authors [95,97], who encountered lower mortalities in gilthead seabream infected with *Photobacterium damselae* subs. *piscicida*, when fed a diet with 5% *Gracilaria gracilis* biomass. Interestingly, the in vitro studies did not reveal a direct inhibitory effect against the pathogen, and this highlights that the result is probably due to the prebiotic capacities of the macroalgae; the pathogen abundance reduction is an indirect effect of the microbial network modulation. More studies are needed to address this potential disease resistance with microbiota modulation by macroalgae, linking microbiota abundance with physiological outputs.

The effects of hydrolysates as protein sources in the gut microbiota have been assessed, but not yet markedly (Table 1). Most studies have focused on the effects of fishmeal or plant protein replacement by hydrolysates together with other alternatives, hardening the isolation of the ingredient contributing for the observed changes. Nonetheless, the available dedicated studies allow us to uncover some microbial modulation patterns, namely the reduction in *Vibrio* genus members abundance or dominance in gut communities. When sardine silage hydrolysate was included at 10 or 19% in European seabass larvae diets, despite the similar *Vibrio* spp. counts when compared with a commercial fish protein hydrolysate, there was a reduction of the genus members’ dominant occurrences [98]. However, in some cases, the *Vibrio* sp. TYH3 strain was still found dominant in fish fed diets with sardine hydrolysate inclusion, and the authors concluded that this ingredient might promote a favourable environment for this *Vibrio* strain, which has opportunistic characteristics. Further, the authors linked this dominance occurrences with the observed higher larval resistance to *Vibrio anguillarum* challenge. Feeding juvenile turbot (*Scophthalmus maximus*) for eight weeks with diets containing up to 34% of enzymatic hydrolysates from poultry by-products resulted in a higher abundance of *Phyllobacterium*, *Sphingomonas*, and *Delftia* genera members [99]. However, this alternative protein was not well-received by fish, resulting in lower digestibility and higher FCR. A possible explanation relies on the fact that the modulated genera are frequently linked to the degradation of aromatic compounds, which might result in the accumulation of toxic metabolites in the intestinal tract, affecting gut health and digestion [99]. Nevertheless, the lowest and highest inclusion levels (8% and 34%, respectively) induced the reduction of members of genus *Vibrio* and increase of *Enterococcus*, favouring the abundance of probiotic-related microorganisms. Gilthead seabream that were fed a diet with 5% hydrolysed fish protein for 92 days presented lower gut microbial diversity, with reduced abundances of Alteromonadales and Enterobacteriales, as well as *Pseudoalteromonas* and *Vibrio* [100]. In agreement, the same species, when fed a diet with 7.5% egg white hydrolysate in a low fishmeal and fish oil diet for eight weeks, presented reduced growth performance, and this was linked with an increase in Spirochaetes and Bacteroidetes abundance, as well as a reduction in Firmicutes [101]. On the other hand, including 18% tuna hydrolysate in barramundi diets for seven weeks improved survival and intestinal histomorphology, with a marked reduction of *Vibrio* members and increased abundance of *Psychrobacter* [102]. The latter have the capacity to secrete essential fatty acids and metabolites that will support host immune and antioxidant status [103], and this might be linked to the observed improved performance. However, data are still very limited, and only few of the published studies have used next generation sequencing (NGS) approaches. Considering the increasing awareness for reducing consumption and introducing circular economy in livestock production, the hydrolysis of waste products has gained much attention [104], and more studies are expected in the short coming future.

A similar trend is expected regarding single cell protein sources. These ingredients include bacteria, fungi (yeast), microalgae-derived products (although, for the purposes of the present review, microalgae are considered a separated alternative protein source together with macroalgae), or combinations, and they are used as protein and omega-3 sources, as well as a source of bioactive compounds (for further information on the different applications of single cell ingredients, see review [16]). From the few available studies on the effects on microbiota, it is possible to infer that those results are not always concomitant. The inclusion of 15% of baker’s yeast biomass in European seabass diets for 93 days had no effect on gut microbial community richness or diversity, but growth performance was enhanced [87]. Interestingly, a lower abundance of members of the families Pseudomonadaceae, Lactobacillaceae, and Pasteurellaceae indicated that changes are directed to neither possible pathogens nor beneficial bacteria, but to a complex orchestration of abundances modulation. A lower gut microbiota diversity in gilthead seabream that were fed diets with 5% autolyzed yeast for 92 days was reported, but the abundance of members of the orders Bacillales and Clostridiales increased, whereas *Pseudoalteromonas* genus members’ abundance decreased, indicating a potential enrichment of beneficial bacteria [100]. The use of yeasts, as a protein source, seems to have a limit of inclusion that is not deleterious for fish. The replacement of 20% of fishmeal with a mixture of yeasts (corresponding to 10% inclusion) in rainbow trout diets had no deleterious effect on gut microbial diversity and composition [105]. In agreement, including up to 10% torula yeast (*Cyberlindnera jadinii*) biomass to replace fishmeal in freshwater Atlantic salmon diets did not affect gut microbial diversity [106]. However, the 20% inclusion of this yeast in a plant protein-based diet reduced growth performance, in association with a reduction in microbial diversity; overall, a higher protein replacement with yeast biomass led to a reduction of LAB abundance in the gut. Further research is needed to properly address the effects of single cell protein sources in gut microbiota, as well as its inclusion limits, covering the same sources in different species, so that robust conclusions can be drawn.

**Table 1:** Summary of alternative proteins (algae, hydrolysates, and yeast-based) and their impacts on fish intestinal microbiota community.

Protein Ingredient	Animal Model and Size	Inclusion Level (%)	Trial Duration	Biological Effects		References
				Microbiota	Relevant Performance, Physiological, and Feed Utilization Output	
**Algae**						
*Ascophyllum nodosum*	Atlantic cod (*Gadus morhua*) 123 g	10	12 weeks	↓ Diversity ↑ *Psychromonas*, *Propionogenium*, and *Clostridium* genera	↓ FBW	[88]
*Arthrospira* (formerly *Spirulina*) *platensis*	European seabass (*Dicentrarchus* labrax) 19 g	15	93 days	↔ Richness and diversity ↓ Proteobacteria ↑ *Persicirhabdus*, *Methylobacterium*, *Acinetobacter*, and *Sediminibacterium* genera	↓ FBW ↑ FCR	[87]
*Arthrospira platensis*	Hybrid grouper (*Epinephelus fuscoguttatus* × *E. lanceolatus*) 28 g	5, 15, 30	47 days	↑ Diversity (Simpson index) in IL5 and IL30; *Vibrio* genus in IL15 ↓ *Tolumonas* genus in IL15	↔ SGR in IL15, and IL30 ↑ SGR in IL5	[92]
*Gracilaria cornea*	Gilthead seabream (*Sparus aurata*) 14 g	5, 15, 25	70 days	↑ Richness; *Pseudomonas* in IL15; *Lactobacillus* in IL25 ↓ *Vibrio*, loss of *Photobacterium* genus as dominant	↔ FBW	[94]
*Schizochytrium limacinum*	Rainbow trout (*Oncorhynchus mykiss*) 31 g	5	15 weeks	↑ Diversity, LAB	↔ FBW, K	[91]
*Ulva ohnoi*	Senegalese sole (*Solea senegalensis*) 11 g	5	45 days	↔ Richness ↑ Diversity in AI, *Vibrio* genus ↓ *Escherichia* and *Tenacibaculum* genera	↓ FBW, WG	[93]
*Ulva pertrusa*	White-spotted rabbitfish (*Siganus canaliculatus*) 15 g	10	8 weeks	↑ *Ruminococcus*, *Clostridium*, and *Lachnospiraceae* genera	↔ FBW, SGR	[107]
*Ulva rigida*	Atlantic cod (*Gadus morhua*) 123 g	10	12 weeks	↔ Community composition	↔ FBW	[88]
*Ulva rigida*	Gilthead seabream (*Sparus aurata*) 14 g	5, 15, 25	70 days	↑ Richness in IL15; *Lactobacillus* and *Sphingomonas* in IL5 and IL15 ↓ Richness in IL25	↑ FBW in IL25	[94]
*Ulva rigida*	Gilthead seabream (*Sparus aurata*) 14 g	25	30 days	↑ Richness, *Lactobacillus brueckii* ↓ *Photobacterium* genus	↑ SR during challenge with *Photobacterium damselae* subs. *piscicida*	[95]
*Tetraselmis chuii* or *Phaeodactylum tricornutum*	Gilthead seabream (*Sparus aurata*) 50 g	10 alone 10 w/*Bacillus*	30 days	↓ Richness and diversity	↑ Intestinal epithelial damage	[90]
**Hydrolysates**						
Fish protein	Gilthead seabream (*Sparus aurata*) 122 g	5	92 days	↔ Richness ↓ Diversity (PD whole tree), Alteromonadales and Enterobacteriales, *Pseudoalteromonas* and *Vibrio* genera	↔ FBW, SGR, FER	[100]
Sardine	European seabass (*Dicentrarchus labrax*) larvae 8 DPH	10, 19	25 days	Changes in *Vibrio* abundance (limited, due to method)	↓ FBW ↑ SR, when challenged with *V. anguillarum*	[98]
Tuna	Barramundi (*Lates calcarifer*) 2.5 g	18	7 weeks	↓ *Vibrio* ↑ *Psychrobacter*	↑ SR, histomorphology	[102]
Egg white	Gilthead seabream (*Sparus aurata*) 20 g	7.5	8 weeks	↔ Diversity ↑ Spirochaetes, Bacteroidetes ↓ Richness, Firmicutes	↓ FBW, K, FI, SGR	[101]
Feather meal	Atlantic salmon (*Salmo salar*) 305 g	20	12 weeks	↔ Dominant bacteria ↑ Abundance of allochtonous bacteria (families Corynebacteriaceae, Lactobacillaceae and Streptococcaceae), Pseudomonadaceae in PI ↓ Vibrionaceae in PI	↔ FBW, SGR	[108]
Poultry by-products	Turbot (*Scophthalmus maximus*) 10.3 g	8, 16, 25, 34	8 weeks	↑ *Phyllobacterium*, *Sphingomonas*, and *Delftia* with increasing IL; *Enterococcus* in IL34; fat synthesis by microbiome in IL25 (predicted) ↓ *Vibrio*	↓ Diet digestibility ↑ FCR	[99]
**Yeasts**						
Yeast (*Saccharomyces cerevisiae*)	European seabass (*Dicentrarchus labrax*) 19 g	15	93 days	↔ Richness and diversity ↓ Pseudomonadaceae, Lactobacillaceae, Pasteurellaceae families ↑ *Persicirhabdus*, *Methylobacterium*, and *Sediminibacterium* genera	↑ FBW	[87]
Yeast	Gilthead seabream (*Sparus aurata*) 122 g	5	92 days	↔ Richness ↓ Diversity (Shannon index), *Pseudoalteromonas* ↑ Bacillales and Clostridialesorders	↔ FBW, SGR, FER	[100]
Torula yeast (*Cyberlindnera jadinii*)	Atlantic salmon (*Salmo salar*) 1.14 g	10, 20	35 days	↓ Diversity in PP diets; LAB in IL20	↓ SGR in PP diets in IL20	[106]
Mixture of yeasts (*Saccharomyces cerevisiae* and *Wickerhamomyces anomalus*)	Rainbow trout (*Oncorhynchus mykiss*) 93 g	10, 20, 30	10 weeks	↔ Diversity in IL10 ↓ Diversity in IL20 and IL30; LAB in IL30 ↑ *Candida albicans*	↔ FBW, FCR	[105]

↔ Without differing from control; ↓ significantly lower than the control; ↑ significantly higher than the control; AI = anterior intestine; FBW = final body weight; FCR = feed conversion ratio; FER = feed efficiency; FI = feed intake; IL = inclusion level; K = condition factor; LAB = lactic acid bacteria; PI = posterior intestine; PP = plant protein-based diets; SGR = specific growth rate; SR = survival rate; WG = weight gain.

The utilization of insect meal for fishmeal and plant protein replacement in aquaculture has been recently addressed by many researchers, and several studies on the effects of this protein source on fish gut microbiota are available. Among the studied insects, the black soldier fly is undoubtably the most studied (Table 2), followed by the yellow mealworm. These ingredients are highly available for a broader usage, and studies have shown an overall improvement in gut health and associated microbiota. Insects are generally known to improve health [109]; considering the results of most studies, this might be due to the effect of microbiota. Within the insect’s composition, chitin is the most relevant and abundant compound. This mucopolysaccharide polymer is composed of ß-1,4-linked N-acetylglucosamine units; since it is not degraded by fish, it is considered an insoluble fibre with potential prebiotic properties [110,111]. Additionally, chitin and its deacetylate derivate, chitosan, are known for having antimicrobial effects on several Gram-negative bacteria [111], and this might be the preferred mode of action for the exerted effects. Although some authors have reported no effect of insect meal on gut microbiota community diversity or richness [87,112,113,114], the majority indicates an increase of diversity [102,115,116,117,118,119,120]. The increase in LAB abundance is one of the most common features that insect meal produces in fish gut microbiota [116,117,118,120,121,122,123,124]. Nevertheless, feeding a diet with 10% hydrolysed yellow mealworm or superworm (*Zophobas morio*) meals reduced *Lactobacillus* and *Carnobacterium* abundance, although no differences in growth were observed. These and other LAB are, in general, regarded as beneficial bacteria that inhibit the growth of pathogenic microorganisms, through the secretion of inhibitory substances, such as bacteriocins and hydrogen peroxide, among others, acting as immunostimulant and improving host’s health, in addition to playing a relevant role in degrading indigestible carbohydrates, such as fibre and starch (see review [76]). In addition to LAB, others are modulated by insect meal and linked with fish health. In Atlantic salmon that were fed a diet with 15% black soldier fly meal for 16 weeks, an increase in the abundance of *Brevinema andersonii* and Spirichaetaceae members was found, and the authors linked those changes with the expression of genes related to immune response and gut barrier function [115]. In rainbow trout, the dietary inclusion of black soldier fly prepupae meal (up to 20%) increased the abundance of *Mycoplasma*, a microorganism that produces lactic and acetic acids as major metabolites, with beneficial consequences for the gut mucosa [119]. Additionally, an increase in *Psychrobacter* abundance was reported in barramundi that were fed a diet with 18% inclusion of black soldier fly meal for seven weeks [102]. The members of this genus produce (and make available) essential fatty acids and metabolites that enhance the host’s immune and antioxidant status [89].

Reports have also indicated that microbiota modulation is dependent not only on fish species, but also on the insect species used as ingredient. The effect of 50% inclusion of yellow mealworm in gilthead seabream, European seabass, and rainbow trout was compared, and differential effects were reported [113]. Microbial diversity remained unchanged in gilthead seabream and European seabass, whereas, in rainbow trout, the Simpson’s diversity index was higher. Moreover, in gilthead seabream, the ratio Proteobacteria:Firmicutes increased, whereas the Firmicutes:Bacteroidetes reduced, and the same was not observed for the other species, indicating highly variable modulation, depending on fish species. However, in this study, fish had different sizes (105, 5, and 115 g for seabream, seabass, and trout, respectively), the feeding trials had different durations (163, 70, and 90 days, respectively), and these differences might harden the comparisons. In another study, the effects of the yellow mealworm, black soldier fly, and housefly (*Musca domestica*) meals, included at 30%, were compared in gilthead seabream and European seabass diets for three months, and different effects on microbiota were also found, depending on fish and insect species [124]. In European seabass, insect meals (regardless the species) increased the abundance of *Anaerococcus*, *Cutibacterium*, and *Pseudomonas*, whereas, in gilthead seabream, the increase in *Staphylococcus*, *Hafnia*, and *Aeromonas*, as well as the reduction of members of Firmicutes phylum, was noticeable. On the other hand, the Firmicutes:Bacteroidetes ratio increased in European seabass that were fed with yellow mealworm and black soldier fly meals, and the same was true when gilthead seabream were fed a diet with housefly meal [124]. In rainbow trout, the effects of four different insect meals (the black soldier fly, yellow mealworm, Indian house cricket (*Gryllodes sigillatus*), and Turkestan cockroach (*Blatta lateralis*)), at 20% dietary inclusion, were tested for 71 days [121]. The authors found that yellow mealworm and cockroach meals improved growth, whereas black soldier fly meal had no effect, and the cricket meal reduced the performance. Although there was higher abundance of LAB (mainly *Lactobacillus* and *Enterococcus*) in all groups, in the cockroach meal-fed group, an increase in *Clostridia coccoides* abundance was observed. This microorganism is linked with the maintenance of gut function, and it might be a reason for the higher villus height observed in this group, which is a positive indicator [121]. One more factor that sums to the complexity in assessing the effects of insect meals in gut microbiota is the insect life cycle stage. Although most studies were performed with insect larvae as a biomass source, different insect stages and processing will affect gut microbial modulation. In rainbow trout juveniles, the effects of black soldier fly meal, when used as defatted larvae, larvae, or prepupae, were compared for five weeks, at a 30% dietary inclusion level [117]. Although insect meal increased microbial diversity, richness, and LAB’s abundance, a higher abundance of *Corynebacterium* genus and Bacillaceae was found in the groups that were fed diets with insect larvae, but not with prepupae. Noticeably, the increase in *Corynebacterium* members—a genus known for producing lipases—occurs in groups fed with diets with high lipidic content, supporting a strong correlation between microbiota and dietary nutritional sources.

From the above-mentioned studies, the overall positive effect of insect meal on fish gut microbiota and health is evident; however, further studies should focus on unravelling the relationships between microbial modulation and physiological outputs, as well as responses and survival when facing insults, such as stress or an infection.

## 4. Impacts of Alternative Protein Sources on Immune Function and Disease Resistance

An activated immune system has specific nutrient requirements and can increase the competition between the nutrients available for maintenance purposes, for the functioning of the immune system, and for body protein deposition in growing animals. This takes particular importance for the aquaculture industry, where nutrition can have significant health implications for fish. Therefore, best practices on diet formulation are of major importance, as feeds represent the leading expenditure to the aquaculture industry [126]. However, keeping in mind the most recent trends in feed formulation for the use of alternative protein sources to decrease the dependency of fishmeal, it is fundamental to evaluate the implications of this new paradigm for fish immune function and disease resistance.

Plant protein ingredients are among the alternatives to fishmeal most studied in the last couple of decades, as a strategy to contribute to aquaculture sustainability and improve cost-effective production. However, most plant protein ingredients have a wide variety of antinutritional factors, which may interfere with fish performance and health, including alterations on immunity, inflammation process, and lower resistance to diseases. Fishmeal replacement, by a blend of plant proteins (i.e., wheat gluten, soybean meal, and soy protein concentrate), negatively affected the immune status of the turbot juveniles fed the highest inclusion levels of plant proteins (>50% of inclusion) [127]. Similarly, fishmeal replacement, by a mixture of plant proteins (i.e., soybean meal, wheat meal, wheat gluten, and corn gluten), in the diets of European seabass decreased plasma immunoglobulins, blood monocytes, and gut interleukin-10 (IL-10) mRNA expression [128]. A five-months feeding trial with low dietary fishmeal levels in European seabass also led to detrimental immune responses [129]. For instance, an up-regulation of IL-1β, tumour necrosis factor α (TNFα), major histocompatibility complex-II (MHCII), and cyclooxygenase-2 (COX-2) was observed, whereas the gut-associated lymphoid tissue decreased its capacity to respond to a sub-lethal dose of *Vibrio anguillarum* via anal inoculation. Gilthead seabream that were fed a diet with total fishmeal replacement, by a blend of wheat gluten, broad bean, soybean, pea, and sunflower meals, showed lower expression in the genes related to pro-inflammatory response, such as IL-1β, IL-6, and COX-2, as well as in immune-related genes, such as immunoglobulin (Ig) M, in line with the high mortality rates observed [130]. Likewise, negative health-related impacts in the gut of turbot were reported after feeding high levels of corn gluten meal, inducing enteritis, and decreasing intestinal immunity, by increasing the inflammatory cytokine transcripts IL-1β, IL-8, and TNF-α [131]. High levels of soybean meal inclusion in diets for several fish species have also been documented to trigger intestinal inflammation [132,133,134]. These adverse effects of plant protein inclusion are known to be related to the presence of antinutritional factors. Although many alternatives have been tested to remove antinutrients and enhance plant protein utilization, few studies focused on immune responses. For instance, dietary inclusion of a fermented plant protein concentrate, up to 40%, in diets for juvenile olive flounder did not affect lysozyme activity nor survival after eight weeks of feeding [135]. In another study, *Aspergillus oryzae*-fermented rapeseed meal was included in diets for red seabream (*Pagrus major*), in order to replace 50% of fishmeal. An increase in lysozyme, respiratory burst, and bactericidal activities in red seabream fed fermented rapeseed meal for 56 days was observed, suggesting a better immune response in these fish, compared to the ones fed with non-fermented rapeseed meal [136]. In contrast, hybrid grouper fed soybean meal or fermented soybean meal for 10 days developed enteritis and showed an increase in the expression of pro-inflammatory genes (IL-1β, IL-8, IL-17, and TNFα), as well as a decrease in anti-inflammatory genes (IL-4, IL-10, and transforming growth factor β1—TGFβ1) and immune parameters, such as lysozyme, complement factors C3 and C4, and IgM [134].

Rendered animal by-products, such as blood meal, poultry by-product meal, feather meal, and bone meal, have also been targeted as fishmeal substitutes, due to their nutrient profile and digestibility. Spray-dried blood and plasma proteins have been recognised as high quality feed ingredients for farmed animals and have been reported to enhance innate immunity in fish. In fact, gilthead seabream that were fed diets with 3 and 6% spray-dried plasma from porcine blood for 60 days showed improved innate immune responses by enhancing serum lysozyme and bactericidal activities [137]. Porcine soluble, originating from porcine mucosal tissues, is a by-product of heparin production that has also been tested in diets for fish. In particular, juvenile rice field eel (*Monopterus albus*) were fed spray-dried porcine soluble at 2.5 and 5% dietary inclusion levels for 56 days, which translated to positive effects at the highest dietary level by enhancing serum complement factors (C3 and C4) and lysozyme activity, while improving gut health [138]. Poultry by-product meal is also a cost-effective ingredient to replace fishmeal. Cobia (*Rachycentron canadum*) juveniles that were fed diets with up to 60% of poultry by-product meal for 10 weeks did not show any changes in haematocrit, haemoglobin, red blood cell, and total immunoglobulins, compared to fish fed a fishmeal-based diet [139]. In contrast, the replacement of fishmeal by chicken plasma powder in diets for largemouth bass negatively affected innate immunity. While inclusion levels of chicken plasma powder at 5 and 10% did not change lysozyme, classical complement pathway, and respiratory burst activities after 12 weeks of feeding, compared to control specimens, a drop in those immune parameters was observed in largemouth bass that were fed diets with 15% of chicken plasma powder [140]. Similarly, total substitution of fishmeal, by poultry by-product meal, negatively influenced the liver health, histological traits of different organs, and immune response in juvenile barramundi after six weeks of feeding [55]. An innovative study tested the effects of a blend of poultry by-product, shrimp, and spray-dried blood meals in diets of juvenile hybrid grouper for eight weeks. At the end of the feeding trial, high levels of dietary processed animal proteins induced hepatic steatosis in hybrid grouper by modulating lipid metabolism-related genes, hepatocyte apoptosis via the up-regulation of apoptosis-related genes, and triggered an inflammatory response via up-regulation of inflammatory cytokines, thus suggesting that hybrid grouper immunity could be impaired, to some extent, by feeding high levels of a blend of processed animal proteins [58]. A recent study assessed the effects of partial replacement of plant proteins by poultry by-product meal (30 and 60% substitution), or in combination with black soldier fly meal, on the inflammatory and immune biomarkers of rainbow trout juveniles fed for 91 days. At the end of the feeding trial, IL-1β, IL-10, TGFβ, COX-2, and T-cell receptor β (TCRβ) gene expression levels in both intestine and head kidney suggested there were no signs of inflammation, and the study concluded that both insect and poultry by-product meals appear to be valid protein sources in fishmeal-free diets [123]. The latter approach of combining poultry by-product and black soldier fly meals was also assessed in barramundi juveniles fed for six weeks. While total fishmeal replacement, by both protein sources, induced detrimental effects at the end of the feeding trial, diets with 55% of fishmeal replacement, by a blend of poultry by-product and insect meals (i.e., 31 + 12% of inclusion, respectively), allowed for enhanced survival against *Vibrio harveyi* infection, in line with an increase in serum lysozyme activity and relative expression of complement factors C3 and C4 in the head kidney [56].

Insects have more recently been considered an appropriate protein source for fish diets, and research efforts are also being directed to study host immune responses, since insect chitin could contribute to boost innate immunity. A recent study with rainbow trout juveniles reported positive effects of black soldier fly larvae inclusion at 8 and 16% in diets with high levels of soybean meal. In this study, insect meal was effective in preventing enteritis by lowering the expression of prostaglandin and interferon regulatory factor 1 in the gut, while the highest inclusion level also improved serum lysozyme activity [33]. In pre-smolt Atlantic salmon, the dietary inclusion of black soldier fly larvae meal and paste were assessed as substitutes of fishmeal and plant proteins during a seven-week feeding trial [141]. While a moderate inclusion of black soldier fly larvae meal (i.e., 12.5%) augmented plasma lysozyme levels and tended to improve the phagocytic activity in head kidney cells, the highest inclusion level (i.e., 25% of insect meal) increased interferon γ and reduced IgM in the distal intestine. In contrast, an inclusion of 35% of black soldier fly larvae paste improved the distal intestine histology. This study also showed minor effects on the expression profile of proteins in skin mucus and no effects on immune markers in splenocytes by the dietary inclusion of black soldier fly larvae meal and paste. Additionally, in Atlantic salmon, another study assessed the effects of total or partial (66%) fishmeal replacement, by black soldier fly larvae meal, on isolated head kidney leukocytes, after simulating an exposure to bacterial or viral antigens. While inflammatory-related gene expression in head kidney cells was not affected by dietary treatments, Atlantic salmon fed with insect meal presented a decrease in the expression of LOX5, a gene coding for an enzyme responsible for the biosynthesis of inflammatory mediators [142]. A study performed with hybrid tilapia (Nile × Mozambique; *Oreochromis niloticus* × *O. mozambicus*) approached the suitability of dietary inclusion of frass, a by-product of the black soldier fly larval meal industry that includes larval excrement, exoskeleton sheds, and residual feed ingredients, along with abundant nutrients, chitin, and beneficial microbes [143]. In this study, frass was included (up to 30%) as partial replacement of a soybean meal, wheat short, and corn meal blend; at the end of the feeding trial (i.e., 12 weeks), the serum complement activity increased significantly in the hybrid tilapia fed the highest frass inclusion level (30%). Moreover, dose-dependent trends in survival, against both *Flavobacterium columnare* and *Streptococcus iniae* challenges, were observed, suggesting that dietary frass from black soldier fly larvae could be beneficial by improving innate immunity and disease resistance of hybrid tilapia against bacterial infection. In an innovative study, black soldier fly larvae were injected with bacteria to boost insect immunity and then incorporated in diets for rainbow trout [144]. Dietary treatments included fishmeal replacement by 25 or 50% of non-immunised or immunised black soldier fly larvae meals. While serum lysozyme levels were increased in rainbow trout that were fed both levels of immunised black soldier fly meal, compared to controls and those fed non-immunised insects, extracts from the immunised black soldier larvae showed inhibitory activity against fish pathogenic bacteria.

Recent research also focused on the potential role of the yellow mealworm, given its excellent nutritional and commercial values. For instance, European seabass that were fed a diet with 25% yellow mealworm inclusion for six weeks showed a drop in serum myeloperoxidase and nitric oxide levels, while lysozyme activity and trypsin inhibition augmented significantly [145]. A similar approach in juvenile yellow catfish (*Pelteobagrus fulvidraco*) assessed the effects of partial fishmeal replacement by yellow mealworm (up to 75%) at the end of a five-week feeding trial, as well as 24 h following bacterial (*Edwardsiella ictaluri*) challenge [146]. While plasma IgM levels were augmented in a dose–response manner with the increasing dietary yellow mealworm content at the end of feeding trial, plasma lysozyme activity was enhanced in yellow catfish that were fed yellow mealworm diets at 24 h following bacterial challenge, in line with the observed up-regulation of immune-related genes (e.g., MHCII, IL-1β, and IgM) in these groups. This study also reported an improved survival against *Edwardsiella ictaluri* in fish that were fed the highest yellow mealworm inclusion level, suggesting a great potential of this insect meal as a strategy to improve yellow catfish immune response and disease resistance. A recent study with juvenile olive flounder was designed to replace 20 to 80% of fishmeal by yellow mealworm (13 to 52% of inclusion), as it was a locally available and affordable protein ingredient, during an eight-week feeding trial [147]. Although this study reported an immunostimulatory effect, to some degree, in olive flounder that were fed the highest yellow mealworm inclusion level, the authors suggested lower inclusion levels (i.e., 40% of fishmeal replacement) to avoid side effects in terms of fillet quality.

Beyond using yellow mealworm and black soldier fly meals as novel protein sources, other studies approached the suitability of other insect species in aquafeeds. Low dietary levels of housefly pupae enhanced peritoneal leukocyte phagocytic activity (i.e., 10 days of feeding at 0.75 and 7.5% inclusion levels) and disease resistance of the red seabream against *Edwardsiella tarda* (i.e., 2 months of feeding at 5% inclusion level) [148]. In contrast, housefly maggot meal, as a fishmeal substitute, in diets for juvenile barramundi decreased plasma lysozyme activity when included at 30% during an eight-week feeding trial [149]. Additionally, the effects of diets containing superworm larvae meal (i.e., 15% and 30%) on Nile tilapia innate immunity were studied after 12 weeks of feeding and following challenge with lipopolysaccharide (LPS) [150]. This study showed an increased lysozyme activity in the serum of Nile tilapia that were fed superworm larvae meal, whereas neutrophils and alternative complement activity in serum were also augmented in those groups after LPS-challenge. Indeed, the potential of using insect meal as a fishmeal substitute, while improving immune machinery and disease resistance, is high; further studies should be directed to develop fortified and sustainable aquafeeds with high inclusion levels of insect meals. Another study approached the possibility of including insect meal, originated from *Spodoptera littoralis*, in diets for Nile tilapia juveniles. Since immune parameters were not altered by increasing the level of this insect meal, the study suggested that fishmeal replacement by this novel ingredient (up to 20%) is feasible for Nile tilapia diets [151]. Other novel insect meal sources were also tested in mirror carp (*Cyprinus carpio* var. *specularis*) diets, during an 11-week feeding trial [152]. In this study, silkworm pupae (*Bombyx mori*) and ragworm (*Nereis virens*) meals were used to replace fishmeal at 24–28%. While no changes were observed for haematological parameters or plasma lysozyme activity, the alternative complement pathway was enhanced in mirror carp fed diets with silkworm pupae inclusion, which could be linked to the presence of long-chain polysaccharides in the pupae meal.

Macro- and microalgae have been introduced as an added-value dietary novel commodity in the last years, thus providing essential and bioactive nutrients to farmed fish. Several species have been addressed as natural sources for improving innate immunity, such as *Ulva rigida*, *Gracilaria gracilis*, and *Arthrospira platensis*, among others. For instance, Nile tilapia juveniles were fed diets with 25% inclusion of *Ulva rigida*, *Crassiphycus corneus* (formerly *Hydropuntia cornea*), and *Scenedesmus almeriensis* for 30 days, being that the diet with *Scenedesmus almeriensis* inclusion was able to enhance respiratory burst, alternative complement pathway and lysozyme activities [153]. An increase in the alternative complement pathway was also observed in gilthead seabream juveniles fed a diet with 25% inclusion of *Ulva rigida* for 30 days, which translated to higher resistance against *Photobacterium damselae* subsp. *piscicida* challenge [95]. An improved disease resistance against the same bacterial pathogen was also observed in gilthead seabream juveniles that were fed *Gracilaria gracilis* powder at 5% dietary inclusion after 52 days, a fact that contrasted with the lower plasma lysozyme activity observed in those specimens before disease challenge, compared to their counterparts that were fed the control diet [97]. In a similar study, European seabass were fed diets with 2.5 and 5% inclusion of *Gracilaria gracilis* powder for 47 days, but no significant changes were observed in innate immune parameters, compared to specimens that were fed the control diet [154]. Similarly, Atlantic salmon that were fed diets at 5, 10, and 15% inclusion levels of dillisk (*Palmaria palmate*) for 14 weeks did not show any changes in haematological parameters, or in plasma lysozyme and alternative complement pathway activities [155].

Many studies in higher vertebrates have been providing insights on the potential for the application of microalgae in fish farming. For instance, when two microalgae (*Nannochloropsis gaditana* and *Tetraselmis chuii*) were orally administered to gilthead seabream for two and four weeks, an increase in haemolytic complement activity, phagocytic capacity, and expression levels of β-defensin in the head kidney were observed [156]. In contrast, Senegalese sole post-larvae that were fed *Nannochloropsis* sp., *Phaeodactylum* sp., and *Chlorella* sp. From heterotrophic production for 41 days did not show any changes in innate immune-related parameters [157]. Another study assessed the effects of *Phaeodactylum tricornutum* incorporation in diets for gilthead seabream by two different processes, either as freeze-dried biomass or broken cell wall biomass, for 12 weeks. Fish that were fed the broken cell wall biomass showed an enhanced immune response by up-regulating the alpha-2-macroglobulin in the head kidney, whereas the alternative complement pathway and bactericidal activities were improved in skin mucus [158]. Gibel carp (*Carassius auratus gibelio*) juveniles were fed diets supplemented with 0.4 to 2.0% of *Chlorella* sp. Powder for 60 days, and an increase in plasma immunoglobulins and in the expression level of IL-22 in the liver and head kidney were observed at low inclusion levels [159]. The effects of *Chlorella vulgaris* in diets for Caspian salmon (*Salmo trutta caspius*) were studied after a 60-day feeding trial, and the highest inclusion level was able to improve plasma IgM and the complement factor C4 [160]. Rainbow trout juveniles that were fed *Chlorella peruviana* at 0.25, 0.50, and 0.75% for 107 days showed a dose–response increase in serum lysozyme and alternative complement pathway activities [161]. Another study approached the effects of 15% dietary inclusion of *Chlorella vulgaris* and *Arthrospira platensis*, individually or blended, in Nile tilapia juveniles that were fed for nine weeks and infected with *Aeromonas hydrophila* for seven days [162]. Both sources of microalga and cyanobacteria were able to improve immunity by increasing serum lysozyme and bactericidal activities, which translated in higher survival against a bacterial pathogen. Similarly, European seabass that were fed spirulina (*Arthrospira platensis*) meal at three different levels (1, 2.5, and 5%) of inclusion for 60 days showed an improved survival against *Vibrio anguillarum* in all spirulina-fed groups. These data were in line with the observed enhancement in innate immune parameters (i.e., respiratory burst, lysozyme, and peroxidase activities) and immune-related gene expression (e.g., IL-1β, TNFα, IL-10, COX-2, IL-6, IL-8, and TGFβ) in a dose-dependent manner [163]. Yellow catfish were fed *Oedocladium* sp. and *Tribonema ultriculosum* at 4 and 5% inclusion levels, respectively, for 40 days and submitted to acute hypoxia stress [164]. Both microalgae were able to enhance plasma complement factor C3 levels after acute stress, whereas dietary *T. ultriculosum* improved plasma IgM content, regardless of hypoxic stress. Zebrafish (*Danio rerio*) was used as a model species, in order to study the effects of including *Tetraselmis* sp., *Phaeodactylum tricornutum*, *Chlorella* sp., *Nannochloropsis oculata*, or *Nannochloropsis gaditana* as additives in a soybean meal-based diet on intestinal inflammation and survival after *Edwardsiella tarda* infection [165]. While the number of neutrophils present in the gut dropped in those fish that were fed most microalgae-diets, the zebrafish survival (following infection) was augmented when fed *Tetraselmis* sp. or *Chlorella* sp. diets.

Amino acid supplementation in aquafeeds is a common practice, as a strategy to safeguard the nutritional requirements that were not fully provided by alternative protein sources, thereby ensuring balanced physiological responses, as well as, whenever possible, enhancing/modulating them. Where the immune function is concerned, there are only a few studies focused on amino acids’ immunomodulatory properties and the effects of their inclusion in diets with alternative protein sources. Methionine is an essential sulphur-containing amino acid, which is limiting in aquafeeds with high inclusion levels of plant proteins. Indeed, a reduction in the expression levels of several immune-related genes was observed in European seabass that were fed a plant protein-based diet for 12 weeks (supplemented with methionine at the currently recommended level for the species), compared to fish that were fed fishmeal-based diets [166]. Interestingly, such a limitation was overcome when the same plant protein-based diet was supplemented with methionine, above the requirement level (0.63 and 0.88% of the feed weight). Taurine is naturally derived from cysteine; hence, it is one of the end-products of the transsulfuration pathway that initiates with methionine. Similar to methionine, its abundance in terrestrial plant ingredients is negligible and, together with limited biosynthesis capacity in fish species [167], its supplementation in current aquafeed is imperative for maintenance of normal physiological conditions and growth performances [168,169,170,171]. Accordingly, rice field eel that were fed a plant protein-based diet showed lower lysozyme activity than fish that were fed a fishmeal-based diet, but it was successfully retrieved when taurine was added up to 0.09% (dry weight) to the plant diet [172]. Tryptophan involvement in the immune response has received moderate attention, where the teleost fish species is concerned. The effect of tryptophan supplementation to plant protein-based diets was tested in gilthead seabream for 21 weeks [173]. Fish that were fed the supplemented diet (27% above the requirement, corresponding to 0.75% of protein) showed an accumulation of acute-phase proteins, without compromising growth rate and feed intake. Additionally, in European seabass that were fed extreme plant protein-based diets (0% fishmeal), tryptophan supplementation slightly, but significantly, primed their immune defences, as shown by an induced expression of T-cell markers and pattern-recognition receptors, such as TRL2 [174].

## 5. Impacts of Alternative Protein Sources on Stress Response

Fish perceive insults and environment alterations through the activation of the central nervous system, triggering a stress response [175]. This is a complex, but vital, cascade of processes that allows animals to adjust and adapt to environmental shifts [176]. The underpinnings of the stress response have been thoroughly described (see reviews [175,177,178]) and will not be addressed here, but it is worth mention that it is a high energy-consuming process. Depending on the stressor and its duration, the trade-off normally jeopardises other physiological processes, such as growth and immunocompetence, with consequences in fish health and overall performance (see reviews [175,179,180]). Dietary modulation of the stress response and its effects has been addressed in several studies, and dietary protein has a great impact on how fish respond to challenges. Reports have indicated that the level of protein in diet alters the stress response in fish [181,182]. However, this is not consensual, and other studies have shown opposing results; for example, a reduction of fishmeal and crude protein levels in rainbow trout diet had no effect on the stress response after six weeks of intermittent handling stress (30 s of chasing, followed by 30 s of netted air exposure), based on cortisol, glucose, and lysozyme plasma levels [183].

In general, functional additives have been the most studied for their stress mitigation properties (see review [26] on this subject), and ingredients, such as probiotics or prebiotics, modulate the stress response in a positive manner. Rainbow trout that were fed a diet supplemented with 0.6% yeast (*Saccharomyces cerevisiae*) and held under crowding conditions for 30 days presented a strong mitigation of the stress-induced transcriptional downregulation in the intestinal mucosa [84], mainly on the immune-related genes. On the other hand, when diets were supplemented with 0.5% yeast-derived mannooligosaccharides, the same was not observed, and the immunosuppression during crowding was evident at molecular level. More, another study (with the same species and diets) showed that, under crowding conditions, dietary inclusion of yeast promotes intestinal homeostasis, buffering the apoptotic cascade that is triggered by the chronic stressor [184]. Some of the ingredients with stress buffering functional properties are great sources of alternative proteins (e.g., microalgae and yeasts); however, the practiced inclusion rates are very low and irrelevant, in terms of protein replacement. Studies have reported the effects of yeasts, as an alternative protein source (Table 3), on acute thermal stress [185,186], hypoxia [185], air exposure [187], handling by net chasing [188], and confinement [189], as well as how the yeast inclusion mitigates stress effects, though the results are not always in agreement. Juvenile Nile tilapia (21 g) that were fed diets with up to 7% yeast (*S. cerevisiae*) for 84 days had better growth performance and higher survival to acute heat stress, regardless of the inclusion rate [185]. However, only when the higher supplementation ratio was provided (7%), a positive effect was seen in survival after 24 h at approximately 0 mg/L of oxygen (hypoxia). The inclusion of up to 50% of ethanol yeast—a co-product from bioethanol production—as a protein source in sunshine bass (*Morone chrysops* × *M. saxatilis*) for nine weeks had no effect on plasma cortisol, glucose, lysozyme, or osmolality levels after a two min net-chasing stressor [188]. However, at 50% yeast inclusion, growth performance was lower, and the feed conversion ratio increased, indicating a deleterious effect in fish. In another study, when 21.4% live yeast (*S. cerevisiae*) was included in the rainbow trout diet for six weeks, growth performance was also reduced [186]. However, dietary yeast reduced the transcription of claudin 6, TNFα, and IL-8 in the posterior intestine after a thermal stress (from 11 to 18 °C), indicating an attenuation of a mucosal response, reducing the triggering of an inflammatory response. This is not necessarily a good indication, since it can be considered that this suppression of pro-inflammatory pathways can limit the gut’s ability to defend and result in higher disease susceptibility. When considering yeasts or similar biomasses, the effects exerted on fish seem to be dependent on the ingredients processing. When diets are cold-pelleted to keep the maximum integrity of the ingredients (e.g., viable probiotics), digestibility might be compromised, when compared with diets that are steam- and heat-extruded, and this should be evaluated in future studies.

Algae biomasses are another alternative protein that, due to the rich bioactive composition, are considered potential stress-mitigator ingredients (Table 3). Including 5% spirulina (*Arthrospira platensis*) in the gilthead seabream diet for 30 days lowered plasma glucose after an air exposure of 60 s, despite an unchanged plasma cortisol [187]. Expression of cytochrome C oxidase IV, peroxiredoxin 2, and thermogenin in the head kidney was reduced before stress, whereas IgM and catalase expression increased in the algae-fed group. These genes are immune and stress-related, and this modulation suggests a possible health improvement, supported during the prophylactic period. Encouraging these results, barramundi that were fed spirulina at 7, 14, or 28% for eight weeks had lower plasma cortisol after a 3 h transport stress [190], indicating a higher stress tolerance. However, at higher inclusion levels, growth performance was compromised, suggesting that fish will tolerate and retrieve higher advantage from lower dosages. In additional agreement, the inclusion of defatted *Tetraselmis* sp. CTP4 biomass at 10% in the gilthead seabream (6 g) diet for 61 days reduced cortisol plasma levels after an acute confinement for 15 min [191]. No differences were observed in the final body weight or FCR; therefore, the exerted effects might have a relation to the bioactive properties of microalgae, which promote resistance and health. In terms of macroalgae, promising results have been reported, regarding its effects on the stress response (Table 3). Nile tilapia that were fed diets including *Sargassum aquifolium* biomass (up to 20%) for 56 days presented a different response to high stocking density than the ones fed a regular diet with no macroalgae [192]. In this study, the deleterious effects of the high stocking density were evident in practically all parameters. However, feeding with *S. aquifolium* promoted better growth performance and higher total proteins, lipase activity, levels of complement factor C3, lysozyme, and other immune response parameters, as well as higher levels of antioxidant enzymes, such as catalase and superoxide dismutase, and total glutathione. Interestingly, in some cases, stressed fish that were fed a diet with macroalgae had such a response that they were considered to be in a better state than unstressed fish. The macroalgae biomass was included, at the expense of wheat bran, and the differences in the stress response and overall better health state were attributed to the *S. aquifolium* composition. This macroalgae is rich in flavonoids, triterpenoids, polyphenols, chlorophyll, carotenoids, and alkaloids, thus being a great antioxidant resource with high radical scavenging capacity; it is also rich in phlorotannins and fucoidan, a sulphated polysaccharide with immunostimulatory, antitumor, antivirus, and hepatoprotective properties [193]. In another study, where gilthead seabream were fed a diet with 5% *Gracilaria vermiculophylla* biomass for 34 days, no differences were observed in growth performance. However, fish had better capacity to survive an acute hypoxia event (24 h at 1.3 mg O_2_/L), showing higher cortisol levels but lower levels of antioxidant markers (lipid peroxidation, catalase, and glutathione peroxidase) [194]. Interestingly, a 5% inclusion, but of *Gracilaria* sp. ethanol extraction waste or agar extraction waste, for 59 days had a slight effect on oxidative stress mitigation, after a crowding event of 100 kg/m^3^ for 1 h [195]; however, other parameters, such as cortisol, glucose, and lactate, remained stable. A 5% inclusion of *Ulva lactuca* biomass in gilthead seabream diets for 34 days increased fish resistance to the acute hypoxia (24 h at 1.3 mg O_2_/L), with higher cortisol levels during stress response, but lower lipid peroxidation during recovery [194]. When feeding European seabass with a diet containing 5% *U. lactuca* for eight weeks, an increase in growth performance was evident, as well as a higher survival during the prophylactic stage [196]. However, increasing the inclusion rate to 10 or 15% increased FCR (with 15%) and reduced survival after an air exposure stress (5 min). When compiled, these results indicate the great potential of algae as a mitigator ingredient of stress effects. However, it is evident that responses are species-, ingredient-, and dosage-specific and there is no “one fits all” solution, supporting the need for further research in precision nutrition to nutritionally target fish specific needs.

**Table 3:** Summary of ingredients and their impacts on the growth performance and stress biomarkers of fish.

Ingredient	Model Species	Inclusion Level (%)	Trial Duration	Stress		References
				Stress Type	Main Observations	
**Algae**						
Spirulina (*Arthrospira* *platensis*)	Gilthead seabream (*Sparus aurata*) 169 g	5	30 days	Air exposure (60 s)	↔ CORT ↓ GLU (after stress); LACT; SOD; *coxiv; prdx3; ucp1* (before stress) ↑ IgM; CAT	[187]
Spirulina (*Arthrospira platensis*) (raw or enzyme processed)	Barramundi (*Lates calcarifer*) 9 g	7, 14, 28	8 weeks	Transport (3 h)	↔ WG, FCR in raw IL7, IL14, and in enzyme processed IL7 and IL14 ↑ FCR in raw in IL28 ↓ WG in raw in IL28; CORT in all	[190]
*Gracilaria vermiculophylla*	Gilthead seabream (*Sparus aurata*) 104 g	5	34 days	Acute hypoxia (24 h; 1.3 mg O_2_/L)	↔ FBW; FCR; WG ↑ CORT in hypoxia; SR after hypoxia; CORT in recovery ↓ CORT in normoxia; CAT in hypoxia; GPx in hypoxia; LPO in recovery	[194]
*Gracilaria* sp. ethanolic extract waste and agar waste biomass	Gilthead seabream (*Sparus aurata*) 9 g	5	59 days	Crowding (100 kg/m^3^ for 1 h)	↔ SGR; FCR; CORT; GLU; LACT; LPO; GPx; GR; GST; GSH; LYZ; PRX ↑ AC50 (compared with non-supplemented)	[195]
*Pterocladia capillacea*	European seabass (*Dicentrarchus labrax*) 0.14 g	5, 10, 15	8 weeks	Air exposure (5 min)	↑ FBW in IL5; SR in IL10 and IL15; FCR in IL15; ↑ SR after stress in IL5 and IL10 ↓ FBW in IL10 and IL15	[196]
*Sargassum aquifolium*	Nile tilapia (*Oreochromis niloticus*) 14 g	5, 10, 15, 20	56 days	High stocking density (20 g/L)	↔ FBW in IL15 and IL20 ↑ FBW in IL5 and IL10; SOD in IL10, IL15, and IL20; WG, RGR, TP, LIP, C3, LYZ, IgM, IgA, GSH, CAT, *cat*, *gst*, *il-10* in all ↓ FCR at IL5 and IL10; ALT, AST in all	[192]
*Tetraselmis* sp. CTP4 (defatted)	Gilthead seabream (*Sparus aurata*) 6 g	10	61 days	Acute confinement (15 min)	↔ FBW; FCR ↓ CORT after stress	[191]
*Ulva lactuca*	Gilthead seabream (*Sparus aurata*) 104 g	5	34 days	Acute hypoxia (24 h; 1.3 mg O_2_/L)	↔ FBW; FCR; WG; CORT in normoxia ↑ LACT in hypoxia; CORT in hypoxia; SR after hypoxia; CORT in recovery ↓ LPO in recovery	[194]
*Ulva lactuca*	European seabass (*Dicentrarchus labrax*) 0.23 g	5, 10, 15	8 weeks	Air exposure (5 min)	↑ FBW in IL5; WG in IL5 and IL10; SR in IL5; FCR in IL15 ↓ FCR in IL5; SR in IL15; SR after stress in IL10 and IL15	[196]
**Hydrolysates**						
Milkfish offal unprocessed (MO) or in hydrolysate (MOH)	Brown-marbled grouper (*Epinephelus* *fuscoguttatus*) 2.8 g	5, 15, 25	8 weeks	Chasing (5 min) and agitation in tank	↔ CORT, GLU in all ↑ FCE in MOH IL15 and IL25; WG in all MOH; FBW in all	[197]
**Yeasts**						
Yeast (*Saccharomyces cerevisiae*)	Gilthead seabream (*Sparus aurata*) 169 g	5	30 days	Air exposure (60 s)	↔ CORT; GLU ↓ *prdx3*, *csf-1*, *ucp1* (before stress); LACT, SOD, *coxiv* (after stress)	[187]
Yeast (*Saccharomyces cerevisiae*)	Nile tilapia (*Oreochromis niloticus*) 21 g	3, 5, 7	84 days	Gradual and acute heat (↔ 40 °C) Hypoxia for 24 h (↔ 0 mg/L)	↑ FBW, SGR, PER in all; SR to acute stress in all; SR to hypoxia in IL7 ↓ FCR in all	[185]
Yeast (*Saccharomyces cerevisiae*)	Rainbow trout (*Oncorhynchus mykiss*) 849 g	32	4 weeks	Confinement by netting (1 min)	↔ FAA levels (after stress) ↑ Met; SAR (before stress)	[189]
Yeast (*Saccharomyces cerevisiae*)	Rainbow trout (*Oncorhynchus mykiss*) 129 g	21.4	6 weeks	Thermal stress (11 > 18 °C)	↑ FCR ↓ SGR; WG; *cld6*; *tnfα*; *il-8* (in PI after stress w/yeast)	[186]
Yeast (*Saccharomyces cerevisiae*)	Sunshine bass (*Morone* *chrysops* × *M. saxatilis*) 4.4 g	27, 41, 50	9 weeks	Net chasing (2 min)	↔ CORT, GLU, LYZ, OSM in all ↑ FCR in IL50 ↓ SGR in IL50; FBW, WG in all; SR w/ increasing IL	[188]
Yeasts blend (*Wickerhamamyces anomalus* and *Saccharomyces cerevisiae* in a 70:30 ratio)	Rainbow trout (*Oncorhynchus mykiss*) 849 g	35	4 weeks	Confinement by netting (1 min)	↔ FAA levels (after stress) ↑ Met; SAR (before stress)	[189]

↔ Without differing from control; ↓ significantly lower than the control; ↑ significantly higher than the control; AC50 = alternative complement system; ALT = alanine aminotransferase; AST = aspartate aminotransferase; C3 = complement factor 3; CAT = catalase; CLD6 = claudin 6; CORT = cortisol; COXIV = cytochrome C oxidase IV; CSF-1 = colony stimulator factor 1 receptor; FAA = free amino acids; FBW = final body weight; FCE = feed conversion efficiency; FCR = feed conversion ratio; GLU = glucose; GPx = glutathione peroxidase; GR = glutathione reductase; GSH = total glutathione; GST = glutathione S-transferase; IgA = immunoglobulin A; IgM = immunoglobulin M; IL = inclusion level; IL-8 = interleukin 8; IL-10 = interleukin 10; LACT = lactate; LIP = lipase; LPO = lipid peroxidation; LYZ = lysozyme; Met = methionine; OSM = osmolality; PER = protein efficiency ratio; PI = proximal intestine; PRDX3 = thioredoxin dependent peroxide reductase; PRX = peroxidase; RGR = relative growth rate; SAR = sarcosine; SGR = specific growth rate; SOD = superoxide dismutase; SR = survival rate; TNFα = tumor necrosis factor alpha; TP = total protein; UCP1= thermogenin; WG = weight gain. Abbreviations in lowercase and italic are gene relative expression.

The effect of insect meal in the stress response has been largely overlooked. Despite the intensification of studies regarding the utilization of this alternative protein, only few studies have addressed a possible effect on the stress response (and not in a direct manner). When Atlantic salmon are fed diets with insect meal (black soldier fly larvae meal at 10% and 15% inclusion) for 56 days, LPS-challenged isolated leukocytes showed a lower expression of the stress-related markers heat shock proteins (HSP70 and HSP27) [142]. When zebrafish were fed defatted black soldier fly V instar or prepupae meal (50% inclusion totally replacing fishmeal) for 60 days (from larvae to adults), an increase in the expression of HSP70 and nr3c1 (glucocorticoid receptor) was observed [198]. Interestingly, differential expressions were observed only in juvenile zebrafish, and HSP70 was only modulated by V instar black soldier fly meal, whereas nr3c1 was only modulated by prepupae meal. This is, however, a topic that is foreseen as being trendy in the short coming future. Insect meal, as an alternative protein source, has been vastly studied, and its effects in fish physiology and immune responses have been consistently addressed over the past five years [20,142]. Chitin and chitosan, as the main components of insect exoskeletons, will be present in the diets [199]; not only do they have great potential as immunostimulants, but they have also been seen to improve stress resistance in rainbow trout [200]. Moreover, the positive effects exerted on gut’s microbiota (see Section 3) might provide stability to better cope with the challenges.

## 6. Impacts of Alternative Protein Sources on Oxidative Stress

Oxidative stress has deleterious impacts on the health status, performance, production, and flesh quality of farmed fish. Hence, maintaining or improving oxidative status is essential for normal physiological processes in fish and, consequently, for the cost-effectiveness of aquaculture industries. Nutrition constitutes one of the major modulators of oxidative stress. The dietary intake of a well-balanced diet with antioxidant compounds can strengthen the animal’s redox status and promote animal allostasis. However, feed ingredients can also trigger oxidative stress situations, due to the presence of antinutritional factors, amino acid deficiencies, or even an excess of proteins/lipids. The molecular mechanisms of nutrition to mediate oxidative stress are complex and poorly understood. Therefore, this review aimed to explore how dietary choices exacerbate or dampen oxidative stress in fish.

When considering new protein sources, the assessment of how the nature and level of a dietary ingredient affects the redox status is often neglected, which, in the long-term, might bring negative consequences to the animal health and growth performance. Several novel protein sources have been evaluated for fish diets, such as oilseeds, legumes, microbes, cereals, algae, insects, and animal by-products. This review aimed to gather insight on the effects of alternative proteins to fishmeal in the oxidative status of the fish that are important for aquaculture productions (Table 4). Most of the work in this field has focused on the inclusion of soybean meal (differently processed, e.g., fermented or dehulled) and its derivatives (e.g., protein concentrate). The inclusion of around 14 to 28% of soybean protein concentrates had no negative impacts on the growth performance and oxidative status of starry flounder (*Platichthys stellatus*) [201] and gilthead seabream [202,203]. However, a higher inclusion (≥38%) seems to induce a state of oxidative stress in the latter. An increase in the level of lipid oxidative damage (LPO), with a concomitantly reduction in the activity of the antioxidant enzymes, superoxide dismutase (SOD) and glutathione peroxidase (GPX), was observed in starry flounder. In gilthead seabream, an increase in the activity of glutathione-S-transferase (GST) and glutathione reductase (GR) enzymes was observed. These enzymes are responsible for the detoxification of endogenous or xenobiotic compounds, through the conjugation of glutathione (GSH), and for restoring the intracellular levels of GSH, respectively. On the other hand, the dietary inclusion of soybean protein concentrates (11 to 42%) for large yellow croaker (*Larimichthys crocea*) [204] and dehulled soybean meal (31 to 56%) for red seabream [205] did not significantly impact the overall redox status of the fish—oxidative damage, reactive oxygen species (ROS) production, and antioxidant defences were stably maintained, when compared to fish that were fed a commercial-like diet. The variable results, with the inclusion of soy products in fish diets, might be attributed to differences in species and developmental stage tested, the type of ingredient processing (fermentation, dehulling, and temperature—affecting ingredient digestibility, content of non-starch polysaccharides, phenols, and antinutritional factors), and dietary formulation (amino acid, mineral, and vitamin supplementation). As a possible alternative to soy, other terrestrial plant ingredients have gained attention for aquafeeds, e.g., rapeseed [206], cottonseed [207], rubber seed [208], sunflower, pea, and lupin [209] meals. A dietary inclusion of around 7 to 20% cottonseed meal in tilapia [208] and rubber seed meal in Ussuri catfish (*Pseudobagrus ussuriensis*) [207] did not deteriorate the fish performance and redox status. However, at higher inclusions (33 to 40%), cottonseed meal was shown to decrease fish antioxidant capacity by a reduction of GPX activity and total antioxidant capacity (TAC) and, consequently, increase the lipid oxidation content, which led the fish to lose the dynamic, balanced relationship of the production and elimination of free radicals in the organism [207]. Similarly, a higher dose (40%) of rubber seed meal also led tilapia to a state of oxidative distress and, consequently, poor growth performances [208]. These fish presented an increase in the activity of endogenous antioxidant enzymes (catalase—CAT and GR); however, the overall antioxidant capacity decreased, which seems to imply that tilapia that were fed a high inclusion of rubber seed meal had a higher production of ROS, which can be linked to an increase in cyanide intake (antinutritional factor present in rubber seed meal). The dried distiller’s grains with solubles (DDGS) is a by-product of ethanol production from cereal grains that can also be included in fish diets [210]. Positive results, in terms of performance, were observed with the replacement of dietary cottonseed meal by DDGS (at 19% inclusion) in diets of grass carp (*Ctenopharyngodon idella*). Moreover, fish were able to maintain the same levels of TAC and LPO, with a reduction of GSH and SOD activity. This better oxidative status might be related to the absence of antinutritional factors, when compared to cottonseed meal, which present gossypol, responsible for inducing the production of superoxide and hydrogen peroxide radicals.

Animal by-products, such as meat, blood, and bone meals, or alternative ingredients, such as mussel and insect meals, are good sources of protein with high palatability and low levels of antinutritional factors. However, in Ussuri catfish, an inclusion of mussel meal (between 9 and 36%) potentiated oxidative stress by decreasing the activity of the first line of enzymatic defence mechanism, SOD and CAT, consequently increasing LPO level [211] (Table 4). A similar response was observed in the same species, when they were fed a diet with 8% inclusion of meat and bone meal [211]. Moreover, poultry by-product meal at 70% inclusion in barramundi diets decreased fish growth performance and led to a state of oxidative stress, through an improvement in the level of LPO and decrease in the activity of GPX [55]. The inclusion of yellow mealworm larvae meal (at 25 and 50%) in rainbow trout diets seems to promote redox status of the fish through the stimulation of endogenous antioxidant defences (SOD, CAT, GPX, and GR) and the concomitant reduction in LPO level, which might be explained by the presence of chitin (immunostimulant capacity) [212].

The inclusion of microalgae in diets for fish was recently reviewed [13]. An inclusion of 19% of *Arthrospira platensis* or *Chlorella vulgaris* in African catfish (*Clarias gariepinus*) diets [213] was shown to promote growth performance and increase the activity of antioxidant SOD and CAT in these fish, compared to fish fed microalgae free diet (Table 4). Overall, the inclusion of these diverse protein sources might be a valuable option to replace fishmeal in the diets for fish, without compromising the growth performance or robustness of the animals.

**Table 4:** Summary of alternative protein and their impacts on fish performance, feed utilization indicators, and oxidative stress biomarkers.

Protein Ingredient	Animal Model & Size	Inclusion Level (%)	Trial Duration	Biological Effects		References
				Performance and Feed Utilization	Antioxidant Status	
Canola meal, processed	Nile tilapia (*Oreochromis niloticus*) 4 g	12, 25, 37, 50	5 weeks	↓ FBW, SGR in IL37 and 50 ↔ FBW, SGR in IL12 and 25; FCR, FI in all	↑ *sod*, *cat*, *gpx* ↔ SOD, CAT, GPX, LPO in all	[214]
Cottonseed meal	Ussuri catfish (*Pseudobagrus ussuriensis*) 2 g	7, 13, 20, 27, 33, 40	8 weeks	↓ FBW, SGR, FE, PER in IL27, 33 and 40; FI in IL33 and 40 ↑ VSI in IL20, 33 and 40 ↔ FBW, SGR, FE, PER in IL7, 13 and 20; FI in IL7, 13, 20 and 27; VSI in IL7, 13 and 27; HSI, K, SR in all	↓ GPX in IL40; TAC in IL33 and 40 ↑ LPO in IL33 and 40 ↔ GPX in IL7, 13, 20, 27, and 33; TAC in IL7, 13, 20, and 27; LPO in IL7, 13, 20, and 27; SOD, CAT in all	[207]
Rapeseed meal, fermented	Red seabream (*Pagrus major*) 4 g	14, 28, 42, 56	9 weeks	↓ FBW, SGR, FI in IL56 ↔ FBW, SGR, FI in IL14, 28 and 42 ↔ FCE, PER, HSI, VSI, SR in all	↓ CAT, ROS in IL56; TAC in IL42 and 56 ↔ TAC in IL14 and 28; CAT in IL14, 28 and 42	[206]
Rapeseed meal, fermented	Red seabream (*Pagrus major*) 6 g	12, 23, 34, 46	8 weeks	↓ FBW, SGR, FCE, PER, SR in IL34 and 46 ↑ FBW, SGR in IL12; VSI in IL23, 34 and 46 ↔ FBW, SGR in IL23; FCE, PER, SR in IL12 and 23; K in IL12, 23 and 34; VSI in IL12; FI, HSI in all	↓ TAC in IL23, 34 and 46; ROS in IL23; CAT in IL34 and 46 ↑ TAC in IL12 ↔ ROS in IL12, 34 and 46; CAT in IL12 and 23	[215]
Rubber seed meal	Tilapia (*Oreochromis niloticus x O. aureus*) 5 g	10, 20, 30, 40	8 weeks	↓ FBW in IL40 ↑ FCR in IL40 ↔ FBW, FCR in IL10, 20, and 30; FI, SR in all	↑ GR in IL40; CAT in IL30 and 40 ↓ TAC in IL30 and 40 ↔ SOD, GPX, LPO; TAC, CAT in IL10 and 20; GR in IL10, 20 and 30	[208]
Soybean meal	Gilthead seabream (*Sparus aurata*) 16 g	20, 39, 58	24 weeks	↓ FBW, SGR in IL39 and 58 ↑ FCR in IL39 and 58 ↔ FBW, SGR, FCR in IL20	↑ CAT, SOD, GR, GST in IL58; GPX in all ↔ CAT, SOD, GR, GST in IL20 and 39	[203]
Soybean meal	*Carassius auratus gibelio*♀ × *Cyprinus carpio*♂ 13 g	13, 26, 40, 54, 68	9 weeks	↓ FBW in IL40, 54 and 68; SGR in IL26, 40, 54 and 68 ↑ FCR in IL54 and 68 ↔ FBW in IL13 and 26; SGR in IL13; FCR in IL13, 26 and 40; SR in all	↓ SOD, CAT in IL68 ↔ SOD, CAT in IL13, 26, 40, and 54	[216]
Soybean meal, dehulled	Red seabream (*Pagrus major*) 7 g	31, 39, 48, 56	8 weeks	↑ FBW, SGR in IL31 and 39 ↔ FBW, SGR in IL48 and 56; PER, HSI, VSI, SR in all	↔ TAC, ROS in all	[205]
Soy protein concentrate	Starry flounder (*Platichthys stellatus*) 13 g	14, 28, 42, 56, 70	10 weeks	↓ FBW, SGR, FI, SR in IL56 and 70; FE, PER, K in IL70 ↔ FBW, SGR, FI, SR in IL14, 28 and 42; FE, PER, K in IL14, 28, 42, and 56; VSI, HSI in all	↓ SOD, GPX in IL56 and 70 ↑ LPO in IL42, 56 and 70 ↔ SOD, CAT, GPX in IL14, 28 and 42	[201]
Soy protein concentrate	Large yellow croaker (*Larimichthys crocea*) 10 g	11, 22, 34, 45	8 weeks	↔ SGR, FCR, FI, SR in all	↔ SOD, CAT, GPX, LPO in all	[204]
Soy protein concentrate, supplemented with methionine and phosphate	Gilthead seabream (*Sparus aurata*) 27 g	20, 38, 58	12 weeks	↓ FBW, SGR, FE in IL58 ↔ FBW, SGR, FE in IL20 and 38; FI in all	↑ GST in IL38 and 58; SOD in IL38; GR in IL58 ↔ GST in IL20; SOD in IL20 and 58; GR in IL20 and 38; CAT, GPX in all	[202]
Distiller’s grains with solubles	Grass carp (*Ctenopharyngodon idellus*) 5 g	10, 19	9 weeks	↓ HSI, K in IL19 ↑ FBW, SGR in IL19; FE in all ↔ FBW, SGR, HSI, K in IL10; VSI, SR in all	↓ SOD, GSH in all ↔ TAC, LPO in all	[210]
Mixture of plant protein sources (corn gluten, wheat gluten, extruded peas, rapeseed meal and sweet white lupin)	Gilthead seabream (*Sparus aurata*) 17 g	33, 52, 65	24 weeks	↓ SGR in IL52 and 65; FBW, FI in all ↑ FE in IL33 and 52 ↔ SGR in IL33; FE in IL65; HSI in all	↑ GSH/GSSH, GR, γGT in IL65 ↔ GSH, GR, γGT in IL33 and 52	[209]
Mixture of plant protein sources (soybean and sunflower meals)	African catfish (*Clarias gariepinus*) 51 g	50, 55, 59, 60	4 weeks	Not determined	↓ TAC in IL59 and 60; SOD, CAT in IL60; GSH in IL55 and 59 ↔ TAC in IL50 and 55; SOD, CAT in IL50, 55, 59; GSH in IL50 and 60	[217]
Mixture of plant and animals’ protein sources [fermented soybean meal and scallop by-product blend (3:2)]	Red seabream (*Pagrus major*) 3 g	11, 23, 34, 46	6 weeks	↓ FBW, SGR, FI, FE, K in IL34 and 46; PER in IL46 ↔ FBW, SGR, FI, FE, K in IL11 and 23; PER in IL11, 23 and 34; HSI, SR in all	↓ TAC in all ↔ ROS in all	[205]
Mussel meal	Ussuri catfish (*Pseudobagrus ussuriensis*) 5 g	9, 18, 36	8 weeks	↓ FBW, SGR, and FI in IL36 ↑ VSI in IL9; PER in IL18 and 36 ↔ FBW, SGR, FI in IL9 and 18; FE, PER in IL9; FCR, VSI in IL18 and 36; HSI, K, SR in all	↓ SOD in IL9, 18 and 36; CAT in IL9; TAC in IL18 and 36 ↑ LPO in IL9, 18 and 36 ↔ TAC in IL9; CAT in IL18 and 36	[211,218]
Meat and bone meal	Ussuri catfish (*Pseudobagrus ussuriensis*) 5 g	8	8 weeks	↓ FBW, SGR, FE, PER, K ↑ VSI ↔ FI, HSI, SR	↓ TAC, SOD, CAT ↔ LPO	[211]
Poultry by-product meal	Barramundi (*Lates calcarifer)* 4 g	70	6 weeks	↓ FBW, SGR, FI ↑ FCR	↓ GPX ↑ LPO	[55]
Black soldier fly (*Hermetia illucens*) meal, defatted	Jian carp (*Cyprinus carpio* var. Jian) 35 gi	3, 5, 8, 11	8 weeks	↔ FBW, SGR, FI, FCR, PER, HSI, VSI, K in all	↔ CAT in IL3, 5 and 8; SOD, LPO in all	[219]
Cricket meal (*Gryllus bimaculatus*)	African catfish (*Clarias gariepinus*) 13 g	26, 35	7 weeks	↓ HSI in all ↑ SGR, FI in all ↔ FCR, PER, SR in all	↑ CAT in IL35 ↔ CAT in IL26; SOD, GST in all	[220]
Yellow mealworm (*Tenebrio molitor*) meal	Rainbow trout (*Oncorhynchus mykiss*) 116 g	25, 50	13 weeks	Not determined	↓ LPO in all ↑ G6PD in IL50; SOD, CAT, GR, GPX in all ↔ G6PD in IL25	[212]
*Chlorella vulgaris*	African catfish (*Clarias gariepinus*) 42 g	13, 19	12 weeks	↓ FCR in all ↑ SGR, K in IL19, FBW, PER, HSI in all ↔ SGR, K in IL13; FI in all	↑ SOD, CAT in all ↔ GST in all	[213]
*Arthrospira platensis*(formerly known as *Spirulina*)	African catfish (*Clarias gariepinus*) 42 g	13, 19	12 weeks	↓ FCR in all ↑ FBW, PER, HSI in all ↔ SGR, FI, K in all	↑ SOD, CAT in all ↔ GST in all	[213]

↔ Without differing from control; ↓ significantly lower than the control; ↑ significantly higher than the control; CAT = catalase; FBW = final body weight; FCE = feed conversion efficiency; FCR = feed conversion ratio; FE = feed efficiency; FI = feed intake; G6PD = glucose 6-phosphate dehydrogenase; GPX = glutathione peroxidase; GR = glutathione reductase; GSH = glutathione; GSSH = glutathione disulphide; GST = glutathione-S-transferase; γGT = γ-glutamyl transferase; HSI = hepatosomatic index; IL = inclusion level; K = condition factor; LPO = lipid peroxidation (malondialdehyde or thiobarbituric acid reactive substances); PER = protein efficiency ratio; ROS = reactive oxygen species; SGR = specific growth rate; SOD = superoxide dismutase; SR = survival rate; TAC = total antioxidant capacity; VSI = viscerosomatic index. Abbreviations in lowercase and italic are gene relative expression.

A myriad of peptides, arising from enzymatic hydrolysis of plant (e.g., soy and wheat) and animal proteins, exhibit strong antioxidant capacity, being able to scavenge radicals and other pro-oxidants. Therefore, in the present review, an overlook of the effect of different sources of protein hydrolysates on the growth performance and redox status of fish were summarised in Table 5. Most of the reports have been conducted with the dietary inclusion of hydrolysates from animals’ by-products. An inclusion of porcine plasma (5%) and blood hydrolysate (3%) in gilthead seabream diets was shown to promote growth performance; however, a not so clear effect was observed in the oxidative status [137,221]. In fact, the supplementation of plasma hydrolysates [137] did not seem to alter the overall antioxidant capacity of the skin mucus of gilthead seabream. In addition, the expression of SOD and CAT in the splenocyte primary cell cultures exposed to a bacteria stimulation was not affected. The dietary inclusion of blood hydrolysates [221] in gilthead seabream did not affect the content of lipidic oxidative damage but reduced the activity of some endogenous antioxidant defences (CAT, GR, and GST). This reduction might be attributed to the presence of bioactive peptides, usually present in animals’ hydrolysates, that can modulate the levels of other antioxidants, maintaining the redox allostasis. On the other hand, an inclusion of more than 2% of feather meal hydrolysates was shown to compromise the oxidative status of Pengze crucian carp (*Carassius auratus* var. Pengze) [222]. An increase in the content of LPO and antioxidant defences was observed in the intestine of carp, which led to a negative effect on the morphology of this organ. Other animal source of hydrolysates come from fish and shellfish by-products. The inclusion of tilapia, krill, and shrimp hydrolysates (3 to 5% inclusion) in the diets of red seabream and olive flounder was shown to improve the growth performance of both species [59,223,224]. However, in these studies, the assessment of the oxidative status of the fish was only based on two antioxidant enzymes, which can only give a slight indication that these hydrolysates can modulate the antioxidant defences. The inclusion of stickwater hydrolysates at 10 and 15% was also shown to promote the growth performance of rice field eel, although it did not reflect a better redox status of the fish, which presented same levels of LPO and SOD activity [225]. The use of soy hydrolysates (6 to 23% inclusion) in the diets of yellow catfish [226] and starry flounder [227] did not have an impact on the oxidative damage of both fish. However, a higher inclusion level was shown to disrupt the redox balance in starry flounder and led to an increase in LPO level, leading to a state of oxidative stress [227]. Overall, the inclusion of hydrolysates in aquafeeds can be a viable strategy to promote animals’ growth and health. Even though protein hydrolysates are considered a good source of antioxidant peptides, many of these studies presented few redox status biomarkers. Therefore, a better understanding of the impacts on animal redox status needs to be further elucidated.

The replacement of fishmeal with terrestrial plant ingredients requires the supplementation of essential amino acids, in particular, methionine and lysine, to compensate any dietary amino acid imbalance. In yellow catfish that were fed a soybean meal-based (inclusion of 36%) diet, the supplementation of lysine and methionine allowed the fish to restore the antioxidant capacity to similar levels of the fish that were fed a commercial-like diet. Fish that were fed the supplemented diet presented a significant lower lipid and protein oxidation, concomitant with a higher redox status (GSH, CAT, and GST) [233]. Methionine is recognised by its role in the regulation of the cellular redox environment. Indeed, methionine residues are highly susceptibility to oxidation, and they can directly react with a variety of ROS and generate methionine sulphoxide, which can be reversibly converted to methionine, through methionine sulfoxide reductases. Moreover, it can also indirectly modulate the oxidative status, through the transsulfuration pathway, which leads to the synthesis of other sulphur amino acids, such as cysteine, that are essential to the production of the endogenous antioxidant GSH, as well as the synthesis of taurine. In fact, rainbow trout that were fed a methionine deficient diet (4.1 mg/kg feed) showed lower growth performance and redox status by decreasing GSH content [234]. On the other hand, a reduction of oxidative damage was observed in the same fish, which seems to be explained by a higher damage in the mitochondria, which led to a reduction on the generation of mitochondrial ROS. Moreover, the total replacement of fishmeal by soybean and canola meal in the diet of blunt snout bream (*Megalobrama amblycephala*) juveniles, supplemented or not with lysine, methionine, and threonine, showed that the addition of these amino acids was able to modulate fish antioxidative status. The amino acid supplementation up-regulated Nrf2 (nuclearfactor erythroid 2-related factor 2), an important transcription factor that can induce the transcription of endogenous antioxidant enzyme genes, as well as the endogenous antioxidant GPX expression [235]. Another important amino acid that is crucial for supporting redox homeostasis in the animal’s cell is taurine. Although it is not an essential amino acid, its requirement is high in many marine species [236]. This amino acid is absent in terrestrial plant ingredients; in addition, it acts as a direct scavenger of ROS and stimulates the antioxidant defence cascade. In a study conducted in gilthead seabream that were fed with graded levels of taurine supplementation, fish showed a linear trend to decrease the LPO level and the activity of antioxidant enzymes SOD, GPX, and GR [237]. This seems to corroborate the ability of taurine to directly neutralise oxidants, leading to a reduction of endogenous antioxidant defences.

Therefore, changes in nutritional factors will affect ROS production and, consequently, modify animals’ antioxidant defences capacity. Understanding the impacts that diet formulation shifts may have on animals’ performance and redox status is pivotal to maximise animal biological efficiency and robustness in the aquaculture industry.

## 7. Conclusions

The intestine is the entry point of feed into fish; together with skin, it integrates the first barrier to external insults. In this sense, when replacing dietary fishmeal by alternative protein sources, the intestine is the first organ that might be affected. Research, up until now, has shown that alternative ingredients, such as terrestrial plant proteins, rendered animal by-products, insect meals, algae, and single cell proteins, may affect gut morphology and integrity. These impacts may decrease nutrient digestibility and absorption, induce intestinal inflammation, and increase disease susceptibility. Additionally, given the important role of the intestine in immune function, the inflammatory reactions observed at the intestinal level in fish that were fed alternative diets may result in an impaired immunological response. Associated with this, the role of fish gut microbiota cannot be disregarded. Microbiota assumes a paramount role as a regulator of fish health. The modulation of gut microbial diversity and richness by alternative protein sources seems to depend on several factors, such as protein processing and dietary inclusion level, target fish species and size, and trial duration. Furthermore, dietary protein has a great impact on how fish respond to challenges, and alternative proteins exert modulatory effects on fish stress response and oxidative stress status. From this literature review, it becomes evident that research of novel protein sources often neglects the possible effects on fish stress response and redox status, which, in the long-term, might bring negative consequences to animal health and growth performance. Despite the fact that crystalline amino acid supplementation in diets is frequently practiced, when fishmeal is replaced by plant or alternative protein sources to fulfil fish requirements, amino acids have specific functions that will benefit neural or metabolic adjustments during stressful events. This is a pertinent topic that deserves attention, especially in the case of tryptophan, due to its important role in stress response, as a precursor of serotonin (5-hydroxytryptamine) and melatonin. In the context of fishmeal replacement by novel protein sources, it seems clear that there are still many knowledge gaps, and research is missing on the potential of dietary supplementation of amino acids as stress mitigators and immunomodulators.

Some of the novel protein sources have functional properties and may exert an overall positive modulatory effect on fish gut microbiota and health. From these, insects clearly stand out, due to their immunomodulatory properties, being able to counteract the negative impacts of some plant and rendered animal proteins. Although research on the use of insect meal has proliferated recently, it is still in its infancy. As highlighted in this review, the beneficial effects of insect meal are clearly dependent on the insect species and development stage. Further research is clearly needed to optimise the nutritional composition of the different insect meals and identify the potential of new insect species, so insect meal can be a sustainable aquafeed ingredient and it’s functional properties may be better explored.

As seen in the past for terrestrial plant ingredients, the future of aquafeeds formulation probably relies on the mixture of different protein sources. In this way, it will be possible to take advantage of the nutritional properties of each ingredient, as well as to benefit from the functional properties that some of these alternative sources seem to present, as in the case of the immunomodulatory effects of insects. However, by mixing different protein sources, synergetic or antagonistic effects might be observed. Therefore, more studies are clearly needed to provide additional value to this new generation of aquafeeds. Further research is paramount, in order to unravel the different implications of these novel protein sources on fish health and welfare, as sustainable pillars of future aquaculture growth.

## Figures and Tables

**Table 2:** Summary of insects’ meal and their impacts on fish intestinal microbiota community.

Protein Ingredient	Animal Model and Size	Inclusion Level (%)	Trial Duration	Biological Effects		References
				Microbiota	Relevant Performance, Physiological, and Feed Utilization Output	
**Insects’ Meal**						
Black soldier fly (*Hermetia illucens*)	Atlantic salmon (*Salmo salar*) 49 g	60	8 weeks	↑ Diversity in mucosa-associated community, Bacillaceae family, *Bacillus*, *Actinomyces* ↓ Digesta-associated community	↔ SGR	[115]
Black soldier fly (*Hermetia illucens*)	Atlantic salmon (*Salmo salar*) 1400 g	15	16 weeks	↑ Diversity and richness, *Brevinema andersonii*, Spirichaetaceae	↔ SGR	[115]
Black soldier fly (*Hermetia illucens*)	Barramundi (*Lates calcarifer*) 3 g	18	7 weeks	↑ Diversity (Shannon and Simpson index), *Psychrobacter* ↓ *Vibrio*	↔ FBW, SGR	[102]
Black soldier fly (*Hermetia illucens*)	European seabass (*Dicentrarchus labrax*) 19 g	15	93 days	↔ Diversity and richness ↓ Proteobacteria and Bacteroidetes, Weeksellaceae, and Prevotellaceae ↑ *Paracoccus*	↑ FBW ↓ FCR	[87]
Black soldier fly (*Hermetia illucens*)	European seabass (*Dicentrarchus labrax*) 6 g	30	12 weeks	↔ Diversity and richness ↑ *Anaerococcus*, *Cutibacterium*, *Pseudomonas*, and Firmicutes:Bacteroidetes ratio	↔ FBW, FCR, K	[124]
Black soldier fly (*Hermetia illucens*)	Gilthead seabream (*Sparus aurata*) 30 g	30	12 weeks	↔ Diversity and richness ↑ *Staphylococcus*, *Hafnia*, and *Aeromonas*	↔ FBW, FCR, K	[124]
Black soldier fly (*Hermetia illucens*)	Siberian sturgeon (*Acipenser baerii*) 640 g	15	60 days	↑ *Bacillus*, *Lactobacillus*, and *Enterococcus* Overall positive and strong modulation	↔ FBW, SGR, FCR ↓ Mucosa thickness	[116]
Black soldier fly (*Hermetia illucens*)	Meagre (*Argyrosomus regius*) 18 g	10, 20, 30	9 weeks	↔ Diversity and community composition	↑ Gut histomorphology alterations	[114]
Black soldier fly (*Hermetia illucens*)	Rainbow trout (*Oncorhynchus mykiss*) 178 g	20, 40	78 days	↑ Diversity, structure, and composition (mainly in IL20) ↑ Digesta associated LAB	↔ WG, FCR	[122]
Black soldier fly (*Hermetia illucens*)	Rainbow trout (*Oncorhynchus mykiss*) 200 g	30	5 weeks	↑ Diversity (Shannon index), richness (Chao index), LAB, *Corynebacterium*, Bacillaceae ↓ Proteobacteria:Firmicutes ratio	↔ WG, FI	[117]
Black soldier fly (*Hermetia illucens*)	Rainbow trout (*Oncorhynchus mykiss*) 53 g	20	71 days	↑ LAB (mainly *Lactobacillus* and *Enterococcus*)	↔ SGR, FCR, villus height, mucosa thickness	[121]
Black soldier fly (*Hermetia illucens*)	Rainbow trout (*Oncorhynchus mykiss*) 66 g	10, 20, 30	12 weeks	↔ Richness ↑ Diversity (Shannon and Simpson) in IL20, *Mycoplasma* ↓ *Aeromonas* and *Citrobacter*	↔ FBW, SGR	[119]
Black soldier fly (*Hermetia illucens*)	Rainbow trout (*Oncorhynchus mykiss*) 66 g	10, 20, 30	12 weeks	↑ Diversity (Shannon and Simpson), richness (Chao1), Firmicutes and Actinobacteria, LAB (mainly Leuconostocaceae and Lactobacillaceae), and Actinobacteria (*Actinomyces, Corynebacterium*)	↔ FBW, SGR, FCR	[118]
Black soldier fly (*Hermetia illucens*)	Rainbow trout (*Oncorhynchus mykiss*) 54 g	8, 23, 45 in PP-based diet	12 weeks	↔ Firmicutes:Proteobacteria ratio ↑ Diversity and richness than PP diet (restored) in IL23 and IL45, *Actinomyces*, *Bacillus*, *Dorea*, *Enterococcus*, *Mycoplasma*	↑ Growth performance, mainly in IL8 combined with poultry meal ↔ Gut barrier integrity	[123]
Black soldier fly (*Hermetia illucens*)	Rainbow trout (*Oncorhynchus mykiss*) 100 g	15	131 days	↑ Richness, Firmicutes (mainly *Bacillus*), *Lactobacillus*, and *Bacillus* ↓ Proteobacteria, *Aeromonas* genus	↔ FBW, SGR	[120]
Housefly (*Musca domestica*)	European seabass (*Dicentrarchus labrax*) 6 g	30	12 weeks	↔ Diversity and richness ↑ *Anaerococcus*, *Cutibacterium*, and *Pseudomonas*	↔ FBW, FCR, K	[124]
Housefly (*Musca domestica*)	Gilthead seabream (*Sparus aurata*) 30 g	30	12 weeks	↔ Diversity ↓ Richness ↑ Firmicutes:Bacteroidetes ratio	↔ FBW, FCR, K	[124]
Indian house cricket (*Gryllodes sigillatus*)	Rainbow trout (*Oncorhynchus mykiss*) 53 g	20	71 days	↑ LAB (mainly *Lactobacillus* and *Enterococcus*)	↑ FCR ↓ SGR, villus height, mucosa thickness	[121]
Yellow mealworm (*Tenebrio molitor*)	European seabass (*Dicentrarchus labrax*) 5 g	50	70 days	↔ Diversity	↔ FBW	[113]
Yellow mealworm (*Tenebrio molitor*)	European seabass (*Dicentrarchus labrax*) 6 g	30	12 weeks	↔ Diversity and richness ↑ *Anaerococcus*, *Cutibacterium*, *Pseudomonas*, and Firmicutes:Bacteroidetes ratio	↔ FBW, FCR, K	[124]
Yellow mealworm (*Tenebrio molitor*)	Gilthead seabream (*Sparus aurata*) 105 g	50	163 days	↑ Proteobacteria:Firmicutes ratio ↓ Firmicutes:Bacteroidetes ratio	↔ FBW	[113]
Yellow mealworm (*Tenebrio molitor*)	Gilthead seabream (*Sparus aurata*) 30 g	30	12 weeks	↔ Diversity and richness ↑ *Staphylococcus*, *Hafnia*, and *Aeromonas*	↔ FBW, FCR ↑ K	[124]
Yellow mealworm (*Tenebrio molitor*)	Rainbow trout (*Oncorhynchus mykiss*) 115 g	50	90 days	↑ Diversity (Simpson index)	↔ FBW	[113]
Yellow mealworm (*Tenebrio molitor*)	Rainbow trout (*Oncorhynchus mykiss*) 53 g	20	71 days	↑ LAB (mainly *Lactobacillus* and *Enterococcus*)	↔ SGR, FCR, mucosa thickness ↓ Villus height	[121]
Yellow mealworm (*Tenebrio molitor*)	Rainbow trout (*Oncorhynchus mykiss*) 80 g	20 (full replacement)	22 weeks	↔ Microbial structure	↔ WG	[125]
Yellow mealworm (*Tenebrio molitor*)	Sea trout (*Salmo trutta*) 5 g	10	8 weeks	↓ *Lactobacillus*, *Carnobacterium*	↔ FBW, SGR, FCR	[112]
Yellow mealworm (*Tenebrio molitor*)	Siberian sturgeon (*Acipenser baerii*) 640 g	15	60 days	↑ Probiotic bacteria (not *Lactobacillus*)	↔ FBW, SGR, FCR ↑ Thickness of muscular layer	[116]
Superworm (*Zophobas morio*)	Sea trout (*Salmo trutta*) 5 g	10	8 weeks	↓ *Aeromonas*, *Enterococcus*, *Lactobacillus*, and *Carnobacterium*	↔ FBW, SGR, FCR	[112]
Turkestan cockroach (*Blatta lateralis*)	Rainbow trout (*Oncorhynchus mykiss*) 53 g	20	71 days	↑ LAB (mainly *Lactobacillus* and *Enterococcus*), *Clostridia coccoides*	↔ SGR, FCR ↑ Villus height, mucosa thickness	[121]

↔ Without differing from control; ↓ significantly lower than the control; ↑ significantly higher than the control; FBW = final body weight; FCR = feed conversion ratio; FI = feed intake; IL = inclusion level; K = condition factor; LAB = lactic acid bacteria; PP = plant protein-based diets; SGR = specific growth rate; WG = weight gain.

**Table 5:** Summary of new sources of dietary hydrolysates and their impacts on fish performance, feed utilization indicators, and oxidative stress biomarkers.

Source	Animal Model & Size	Inclusion Level (%)	Trial Duration	Biological Effects		References
				Performance and Feed Utilization	Antioxidant Status	
Feather meal	Pengze crucian carp (*Carassius auratus* var. Pengze) 13 g	2, 4, 6, 8	10 weeks	↓ FBW, SGR in IL8; FE in IL4 ↑ HSI in IL8 ↔ FBW, SGR, HSI in IL2, 4 and 6; FE in IL2, 6 and 8; SR in all	↓ CAT in IL2 ↑ GSH, LPO in IL4, 6 and 8; CAT in IL8; SOD, GPX in all ↔ LPO, GSH in IL2; CAT in IL4 and 6	[222]
Porcine plasma	Gilthead seabream (*Sparus aurata*) 182 g	5	13 weeks	↑ FBW, SGR, FI ↔ FCR, K, SR	↔ TAC, *sod*, *cat*	[221]
Porcine blood	Gilthead seabream (*Sparus aurata*) 1 g	3, 6	9 weeks	↑ FBW, SGR, K in IL3 ↔ FBW, SGR, K in IL6; SR in all	↓ CAT, GR, GST in IL3 ↔ CAT, GR, GST in IL6; LPO, GPX in all	[137]
Krill	Olive flounder (*Paralichthys* *olivaceus*) 15 g	3	11 weeks	↓ FCR ↑ FBW, SGR, FI, PER ↔ SR	↔ SOD, GPX	[59]
Krill	Red seabream (*Pagrus major*) 29 g	4	12 weeks	↓ FCR ↑ FBW, SGR, PER ↔ FI, SR	↑ SOD	[224]
Krill	Red seabream (*Pagrus major*) 5 g	3	13 weeks	↓ FCR ↑ FBW, SGR, PER ↔ FI, HSI, VSI, K, SR	↑ SOD ↔ GPX	[223]
Shrimp	Olive flounder (*Paralichthys olivaceus*) 15 g	3	11 weeks	↑ FBW, SGR, FI ↔ FCR, PER, SR	↑ SOD ↔ GPX	[59]
Shrimp	Red seabream (*Pagrus major*) 29 g	5	12 weeks	↓ FCR ↑ FBW, SGR, PER ↔ FI, SR	↔ SOD	[224]
Shrimp	Red seabream (*Pagrus major*) 5 g	3	13 weeks	↓ FCR ↑ FBW, SGR, PER ↔ FI, HSI, VSI, K, SR	↑ SDO ↔ GPX	[223]
Silkworm pupa	Mirror carp (*Cyprinus carpio* var. *specularis*) 15 g	3, 5, 8, 10	8 weeks	↓ FBW, SGR in IL8 and 10 ↑ FCR in IL8 and 10 ↔ FBW, SGR, FCR in IL3 and 5; PER, VSI, HSI, K, SR in all	↑ SOD in IL3 and 5; CAT in IL3 ↔ SOD, LPO in IL8 and 10; CAT in IL5, 8 and 10	[228]
Tilapia	Olive flounder (*Paralichthys* *olivaceus*) 15 g	3	11 weeks	↓ FCR ↑ FBW, SGR, FI ↔ PER, SR	↔ SOD, GPX	[59]
Tilapia	Red seabream (*Pagrus major*) 29 g	4	12 weeks	↔ FBW, SGR, FI, FCR, PER, SR	↔ SOD	[224]
Tilapia	Red seabream (*Pagrus major*) 5 g	3	13 weeks	↓ FCR ↑ SGR, PER ↔ FBW, FI, HSI, VSI, K, SR	↑ SOD ↔ GPX	[223]
Stickwater	Rice field eel (*Monopterus albus*) 25 g	5, 10, 15	8 weeks	↓ FCR, K in IL10 ↑ FBW in IL10 and 15 ↔ FBW in IL5; K in IL5 and 15; SR, VSI, HSI in all	↔ SOD, LPO in all	[225]
Soy	Yellow catfish (*Pelteobagrus* *fulvidraco*) 22 g	6, 11, 16	8 weeks	↓ FCR in IL16; HSI in IL6 and 11 ↑ FBW, SGR in IL16 ↔ FBW, SGR, FCR in IL6 and 11; HSI in IL16; VSI, SR in all	↔ LPO in all	[226]
Soy	Starry flounder (*Platichthys* *stellatus*) 6 g	14, 23, 38, 50, 62, 73	9 weeks	↓ SGR in IL73; FCR in IL14, 23 and 38; FI, PER in IL62 and 73 ↑ SGR, FI, PER in IL14, 23 and 38; FCR in IL62 and 73 ↔ FBW, SGR in IL50 and 62; FCR, FI, PER in IL50	↓ LPO in IL23, 38, 50, 62, and 73 ↑ TAC in IL14, 23, 38, and 50; SOD in all ↔ LPO in IL14; TAC in IL62 and 73	[227]
Soy, low and high hydrolysis	Rainbow trout (*Oncorhynchus mykiss*) 18 g	7, 14, 21	8 weeks	↓ FBW and WG in IL21 ↑ FCR in IL21 ↔ FBW, WG in IL7 and IL14; FCR, FI, K, HSI, VSI in all	↑ SOD in IL14 and 21 ↔ SOD in IL7	[229]
Whey	Arctic charr (*Salvelinus alpinus*) 34 g	0.1, 0.5, 1.0, 2.6, 5.1	12 weeks	↔ FBW, SGR, FI, FCR, PER, HSI, VSI in all	↔ TAC, GSH, LPO in all	[230]
*Nannochloropsis gaditana*	Siberian sturgeon (*Acipenser baerii*) 13 g	10	6 weeks	↔ FBW, SGR, FI, FCR, K, SR	↔ SOD, CAT	[231]
*Scenedesmus almeriensis*	Siberian sturgeon (*Acipenser baerii*) 13 g	10	6 weeks	↓ FBW ↔ SGR, FI, FCR, K, SR	↔ SOD, CAT	[231]
*Saccharomyces cerevisiae*	Orange-spotted grouper (*Epinephelus coioides*) 10 g	1, 2, 3, 5	8 weeks	↔ SGR, FCR, FI, PER, SR in all	↓ SOD in IL3 and 5 ↔ SOD in IL1 and 2; CAT in all	[232]

↔ Without differing from control; ↓ significantly lower than the control; ↑ significantly higher than the control; CAT = catalase; FBW = final body weight; FCR = feed conversion ratio; FE = feed efficiency; FI = feed intake; GPX = glutathione peroxidase; GR = glutathione reductase; GSH = glutathione; GST = glutathione-S-transferase; HSI = hepatosomatic index; IL = inclusion level; K = condition factor; LPO = lipid peroxidation (malondialdehyde or thiobarbituric acid reactive substances); PER = protein efficiency ratio; SGR = specific growth rate; SOD = superoxide dismutase; SR = survival rate; TAC = total antioxidant capacity; VSI = viscerosomatic index; WG = weight gain. Abbreviations in lowercase and italic are gene relative expression.
